# Apoptosis signal-regulating kinase 1 (*Ask1*) deficiency alleviates MPP^+^-induced impairment of evoked dopamine release in the mouse hippocampus

**DOI:** 10.3389/fncel.2024.1288991

**Published:** 2024-02-13

**Authors:** Fang Zhao, Chuhan Li, Yinghan Zhuang, Yan Yan, Yanqin Gao, Thomas Behnisch

**Affiliations:** State Key Laboratory of Medical Neurobiology and MOE Frontiers Center for Brain Science, Institutes of Brain Science, Fudan University, Shanghai, China

**Keywords:** memory, *Ask1*, hippocampus, dopamine release, Parkinson’s disease, memory decline

## Abstract

The dopaminergic system is susceptible to dysfunction in numerous neurological diseases, including Parkinson’s disease (PD). In addition to motor symptoms, some PD patients may experience non-motor symptoms, including cognitive and memory deficits. A possible explanation for their manifestation is a disturbed pattern of dopamine release in brain regions involved in learning and memory, such as the hippocampus. Therefore, investigating neuropathological alterations in dopamine release prior to neurodegeneration is imperative. This study aimed to characterize evoked hippocampal dopamine release and assess the impact of the neurotoxin MPP^+^ using a genetically encoded dopamine sensor and gene expression analysis. Additionally, considering the potential neuroprotective attributes demonstrated by apoptosis signal-regulating kinase 1 (*Ask1*) in various animal-disease-like models, the study also aimed to determine whether *Ask1* knockdown restores MPP^+^-altered dopamine release in acute hippocampal slices. We applied variations of low- and high-frequency stimulation to evoke dopamine release within different hippocampal regions and discovered that acute application of MPP^+^ reduced the amount of dopamine released and hindered the recovery of dopamine release after repeated stimulation. In addition, we observed that *Ask1* deficiency attenuated the detrimental effects of MPP^+^ on the recovery of dopamine release after repeated stimulation. RNA sequencing analysis indicated that genes associated with the synaptic pathways are involved in response to MPP^+^ exposure. Notably, *Ask1* deficiency was found to downregulate the expression of *Slc5a7*, a gene encoding a sodium-dependent high-affinity choline transporter that regulates acetylcholine levels. Respective follow-up experiments indicated that *Slc5a7* plays a role in *Ask1* deficiency-mediated protection against MPP^+^ neurotoxicity. In addition, increasing acetylcholine levels using an acetylcholinesterase inhibitor could exacerbate the toxicity of MPP^+^. In conclusion, our data imply that the modulation of the dopamine-acetylcholine balance may be a crucial mechanism of action underlying the neuroprotective effects of *Ask1* deficiency in PD.

## 1 Introduction

Dopamine (DA) release in the hippocampus is fundamental for learning, memory, and cognitive function (Schultz, 2002; Rossato et al., 2009; Lisman et al., 2011; Pezze and Bast, 2012). Dysfunction in the dopaminergic system, seen in neurological diseases like Parkinson’s disease (PD), may lead to cognitive and memory deficits in addition to motor symptoms. The hippocampus, located in the medial temporal lobe, plays a crucial role in cognitive processes and memory formation, with extensive connections to other cortical regions (van Strien et al., 2009). Accordingly, activity-dependent synaptic plasticity in the hippocampus, induced by neuronal activity, is a key mechanism underlying memory formation. Activity-dependent synaptic plasticity in the hippocampus is induced by high- or low-frequency stimulation of hippocampal afferents, leading to long-term potentiation (LTP) or long-term depression (LTD) of synaptic transmission, respectively (Bliss and Collingridge, 1993; Sajikumar and Frey, 2004; Reymann and Frey, 2007; Wong et al., 2007; Gong et al., 2009; Larson and Munkácsy, 2015). Hippocampal LTP and LTD have been strongly associated with memory formation (Morris, 2006; Nicholls et al., 2008; Redondo and Morris, 2011).

The hippocampus receives modulatory inputs from various brain regions, including the dopaminergic system, to regulate basal network activity and synaptic plasticity involved in memory formation (Rossato et al., 2009; Lisman et al., 2011). Dopamine, released in the hippocampus, contributes to synaptic plasticity and memory formation through the activation of dopamine D1/D5 receptors, which are abundantly expressed in this brain region (Levey et al., 1993; Ciliax et al., 2000). Studies have demonstrated the involvement of these receptors in converting short-term potentiation (STP) to long-term potentiation (LTP), maintaining LTP, and inducing long-term depression (LTD) (Moncada and Viola, 2007; Rossato et al., 2009; Wang L. et al., 2010; Lemon and Manahan-Vaughan, 2012; Pezze and Bast, 2012).

Dopaminergic neurons, located in the ventral midbrain’s substantia nigra pars compacta (SNc) and ventral tegmental area (VTA), project connections to various brain regions, including the hippocampus and dentate gyrus. The hippocampus-VTA pathway plays a crucial role in memory consolidation, storage, and retrieval (Lisman and Grace, 2005). Within the hippocampus, dopaminergic fibers possess the necessary components for dopamine synthesis, release, and reuptake. However, it has been observed that the distribution of dopaminergic projections in the hippocampus is diffuse and lacks clear colocalization within hippocampal regions and layers, such as well-defined pathways like the mossy fiber or Schaffer collateral pathways. Instead, the distribution appears to be random and lacks the distinct fiber enrichment commonly seen in specific pathways (Chen et al., 2018). Similarly, in the striatum, dopamine release from axonal varicosities has been observed, some of which exhibit active zone-linked proteins like RIM and Bassoon (Liu et al., 2018; Banerjee et al., 2022). These findings indicate that dopamine release may show differences from the recognized dependencies in excitatory glutamate release. Therefore, it is crucial to characterize dopamine release probabilities in response to various activation frequencies in different hippocampal sublayers, such as stratum radiatum (SR) and stratum lacunosum-moleculare (SLM) in CA1 and CA2, commonly used to induce specific forms of synaptic plasticity. In the hippocampus, dopamine (DA) modulates synaptic transmission through two receptor subgroups: D1-like receptors (D1R), which stimulate adenylyl cyclase (AC), and D2-like receptors (D2R), which inhibit AC (Beaulieu and Gainetdinov, 2011). Previous studies have demonstrated that D1R activation mediates presynaptic facilitation (Cameron and Williams, 1993; Radnikow and Misgeld, 1998), while D2R activation mediates presynaptic inhibition (Cooper and Stanford, 2001; Mengual and Pickel, 2002). D1R activation plays a critical role in LTP maintenance in the hippocampus (Sajikumar and Frey, 2004; Swant and Wagner, 2006; Papaleonidopoulos et al., 2018). Conversely, D2R activation can inhibit basal synaptic transmission (Manahan-Vaughan and Kulla, 2003) and timing-dependent LTP (Cepeda-Prado et al., 2022).

Disruption of the dopaminergic system is implicated in neurological diseases such as Parkinson’s disease (PD) and Huntington’s disease. PD involves the progressive degeneration of dopaminergic neurons in the substantia nigra pars compacta (SNc), leading to motor deficits and potentially non-motor disorders like mild cognitive impairment (MCI) and dementia (Kikuchi et al., 2017; Hendrickx et al., 2021). 1-methyl-4-phenyl-1,2,3,6-tetrahydropyridine (MPTP), a useful tool to induce the development of both late and early PD models, can reproduce both motor and non-motor symptoms (Grandi et al., 2018). MPTP is metabolized by the enzyme monoamine oxidase-B (MAO-B) into 1-methyl-4-phenylpyridinium (MPP^+^). MPP^+^ can selectively enter dopamine neurons through the dopamine transporter (DAT), which plays a crucial role in MPTP-induced neurotoxicity (Singer and Ramsay, 1990; Samii et al., 2004). Consequently, researchers have employed MPTP and MPP^+^ to investigate mechanisms of neurotoxicity, specifically targeting the dopaminergic system. In addition to the chronic effects leading to the apoptotic death of dopaminergic neurons in the substantia nigra pars compacta (SNc), these substances can also be used to explore more subtle effects on dopaminergic axons or varicosities. Neuropathological and neurological changes might occur before obvious neurodegeneration, potentially triggered by environmental toxins or genetic factors (Grandi et al., 2018; Hendrickx et al., 2021). Therefore, investigating alterations in dopamine release probabilities caused by low concentrations of the neurotoxin MPP^+^ and exploring protective approaches is an important research objective (Lisman et al., 2017). Consequently, targeting dopamine release pathways that are crucial for memory formation could serve as a therapeutic strategy to alleviate cognitive impairment in patients with PD.

Apoptosis signal-regulating kinase 1 (*Ask1*), a member of the mitogen-activated protein kinase (MAPKK) family, plays a role in regulating inflammation and apoptosis through the c-Jun kinase (JNK) and p38 pathways (Yu and Richardson, 2011). Studies have shown that *Ask1* deficiency improves motor performance in chronic models of Parkinson’s disease (PD) induced by MPTP and in α-synuclein transgenic mice (Lee et al., 2012, 2015). Additionally, *Ask1* has been implicated in cognitive impairment (Toyama et al., 2014), making it a potential target for protecting hippocampal dopamine release from neurotoxins.

To observe the dynamics of dopamine release in acute hippocampal slices, we employed the genetically encoded fluorescence dopamine sensor dLight1.2. dLight1.2 utilizes a cpGFP module from GCaMP6, replacing the third intracellular loop of the human dopamine D1 receptors, to enable precise visualization of dopamine release in various brain regions (Patriarchi et al., 2018). We investigated the protective effects of *Ask1* deficiency against MPP^+^ toxicity using a transgenic mouse strain with a tamoxifen-inducible Cre-lox system. The Cre recombinase is fused to an estrogen receptor (ER), rendering it inactive. Upon administration of tamoxifen, the Cre-ER fusion protein is activated and catalyzes the conditional deletion of the lox-flanked exon 2 of the *Ask1* gene. This prevents *Ask1* expression and generates a mouse model with a conditionally controlled *Ask1* deficiency.

This approach monitored dopamine release dynamics in hippocampal slices upon electrical stimulation, allowing a comparison of genotypes to assess *Ask1* deficiency’s effects on reduced dopamine release caused by MPP^+^. RNA sequencing analyzed molecular changes linked to *Ask1* knockdown and acute MPP^+^ exposure, identifying *Slc5a7* as one of the differentially expressed genes. Previous studies suggest that acetylcholine influences dopamine transmission in the striatum (Rice and Cragg, 2004; Threlfell et al., 2012). To determine if inhibiting *Slc5a7* could mimic *Ask1* deficiency’s impact on impaired dopamine release from MPP^+^, we conducted experiments interfering with this transporter. Thus, we described the impact of *Ask1* deficiency on dopamine release dynamics in the hippocampus, revealing potential neuroprotective effects against MPP^+^ toxicity.

## 2 Materials and methods

### 2.1 Ethics statement

The animal care and procedures were conducted in compliance with the established research standards of the Institutes of Brain Science and State Key Laboratory of Medical Neurobiology of Fudan University, Shanghai. All experimental protocols were approved by the Institutional Animal Care and Use Committee of Fudan University, Shanghai Medical College (approval no. 31320103906).

### 2.2 Animals

Male C57BL/6 mice (6–8 weeks old) were obtained from JieSiJie Company (Shanghai). *Ask1* conditional knock-out mice (*Ask1*^−/−^) were generated by crossbreeding *Ask1*^flox/flox^ (Map3k5^flox/flox^, Shanghai Model Organisms Center) with CAGGCre-ER™ mice (RRID: IMSR_JAX:004682), which were kindly provided by Professor Gao (Institutes of Brain Science, Fudan University). All animals were provided with unrestricted access to a standard diet and water and were kept in housing facilities that maintained appropriate temperatures and humidity, following the recommended standards and procedures of the Institutes of Brain Science and State Key Laboratory of Medical Neurobiology (Fudan University, Shanghai, China). To induce Cre recombination and generate *Ask1*^−/−^ mice, interbred CAGGCre-ER™ + *Ask1*^flox/flox^ mice were administered intraperitoneal (i.p.) injections of 0.1 mL 20 mg/mL tamoxifen (Sigma-Aldrich T5648) once daily for five consecutive days. To prepare the tamoxifen solution, tamoxifen was dissolved in a mixture of corn oil and absolute ethyl alcohol using overnight agitation at 37°C, following the method described by Hayashi and McMahon (2002). After injection, mice were allowed to rest for at least 1 week. The success of the procedure was verified through PCR analysis and western blotting of hippocampal brain tissue upon completion of the experiments, as shown in Supplementary Figure S1.

### 2.3 Adeno-associated virus and stereotaxic surgery

The plasmid pAAV-hSyn-dLight1.2, obtained from Addgene (#111068, Lin Tian Lab), was used to generate adeno-associated virus vectors by Shanghai Shengbo (China). To initiate the transduction process, mice were anesthetized using 2.5% avertin (2,2,2-tribromoethanol) at a dose of 0.02 mL/g through intraperitoneal injection. The anesthetized mice were then positioned in a stereotaxic apparatus. For bilateral injection into the hippocampal CA1 region, the dLight1.2 construct was injected at coordinates of anterior–posterior = −3.5 mm, mediolateral = ±3.6 mm, and dorsoventral = −3.0 mm relative to bregma (Paxinos and Franklin, 2001). The injection rate used was 0.5 μL/5 min. Following each injection, the needle was left in place at the injection site for 5 min to ensure optimal transduction.

### 2.4 Acute hippocampal slice preparation

One to two weeks after transducing hippocampal neurons with pAAV-hSyn-dLight1.2, acute hippocampal slices were prepared from anesthetized mice using isoflurane and decapitation. The brain was quickly harvested and placed in ice-cold NMDG artificial cerebrospinal fluid (aCSF) saturated with a gas mixture of 95% O_2_ and 5% CO_2_ for a few minutes. The NMDA aCSF had the following composition (in mM): 93 NMDG, 2.4 KCl, 1.33 NaH_2_PO_4_·2H_2_O, 20 HEPES, 10 MgSO_4_, 0.5 CaCl_2_, 30 glucose, 30 NaHCO_3_, 5 sodium ascorbate, 3 sodium pyruvate, and 20 HEPES, with a pH of 7.3–7.4. Next, transverse hippocampal slices (350 μm) were obtained using a vibratome (Leica Wetzlar, Germany) and stored in aCSF at 32°C for at least 2 h. Subsequently, the slices were transferred to a recording chamber mounted on a Nikon microscope stage (Nikon Instruments Inc., Melville, NY, USA), with a constant flow of aCSF at a rate of 4 mL/min. The aCSF used for incubation and experiments had the following composition (in mM): 124 NaCl, 2.5 KCl, 2 CaCl_2_·2H_2_O, 2 MgCl_2_·7H_2_O, 1.25 NaH_2_PO_4_, 10 d-glucose, and 26 NaHCO_3_, with a pH of 7.4. This aCSF was used for both fluorescence imaging and field potential recording.

### 2.5 Electric stimulation protocols and field potential recordings

**Figure 1:**
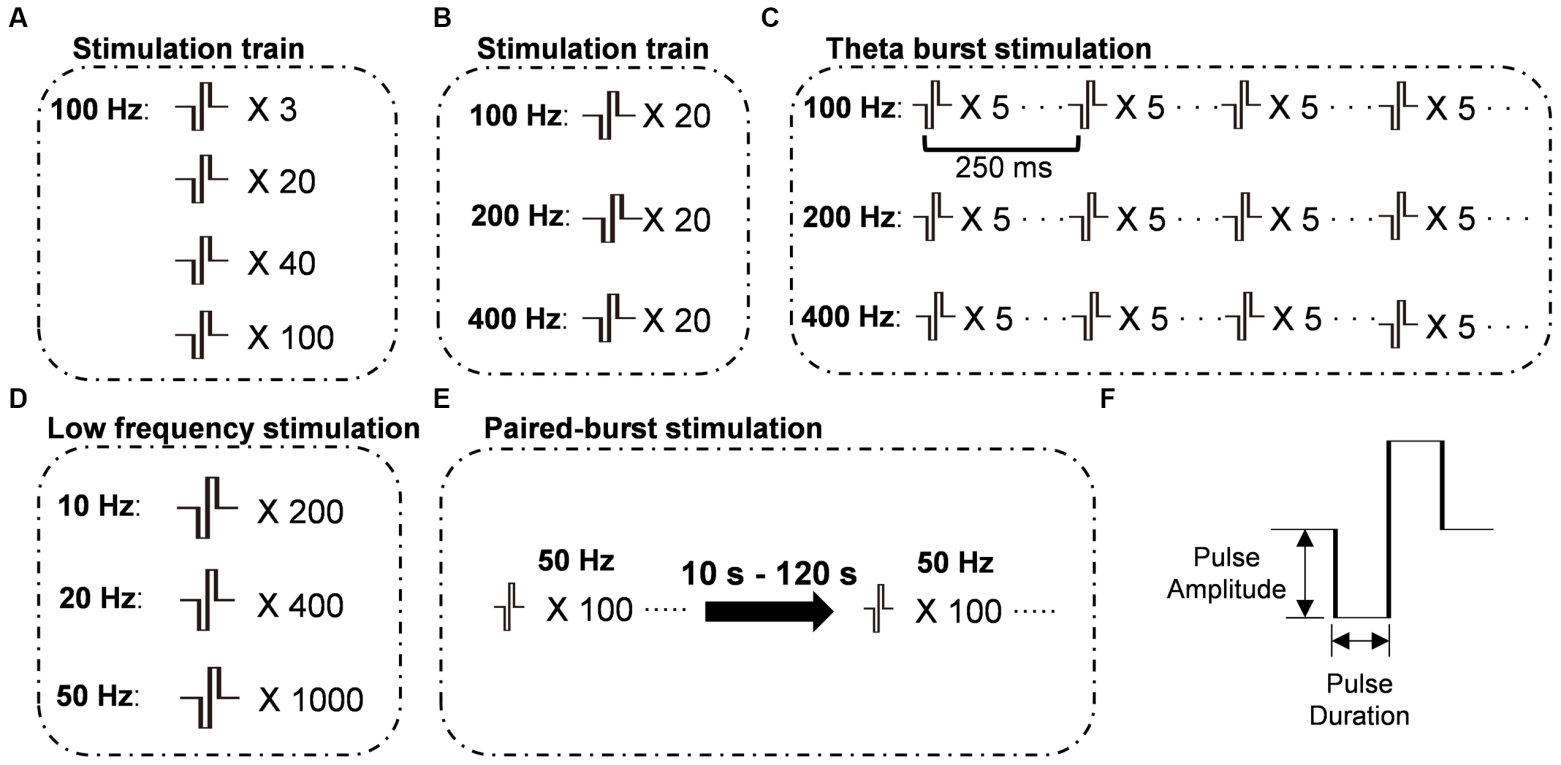
Schematic representations of stimulation protocols. **(A)** Stimulation protocols were administered at 100 Hz with varying numbers of pulses. **(B)** Stimulation protocols were administered with a constant number of 20 pulses, while the frequency of stimulation varied. **(C)** Theta-burst stimulation protocols were administered with varying burst frequencies while maintaining a constant inter-burst interval of 250 ms. **(D)** Train stimulation protocols were administered at frequencies below 100 Hz with varying pulse numbers to maintain a constant total stimulation time. **(E)** Paired-burst stimulation was administered at different inter-burst intervals (IBI) ranging from 10 to 120 s, with two bursts consisting of 100 pulses at 50 Hz. **(F)** The stimulus pulse was a biphasic square wave with a single-phase duration of 0.2 ms. X number: number of pulses; Frequency: frequency of bursts.

Field excitatory postsynaptic potentials (fEPSPs) were recorded using glass pipettes (5–8 MΩ) filled with aCSF (see Figure 1A). The fEPSP recordings were amplified, and filtered using an Axon amplifier (MultiClamp 700B; Molecular Devices, San Jose, CA, United States) with a 0.1-Hz high-pass filter and a 3-kHz low-pass filter, and digitized at a sampling frequency of 20 kHz using an AD/DA converter software system (Digidata 1,400; Molecular Devices, San Jose, CA, United States). To stimulate dopamine release, glass pipettes filled with aCSF were used to electrically stimulate fibers in CA1-SR, CA2-SR, and CA1-SLM. The specific stimulation protocols applied to induce dopamine release are schematically depicted in Figure 1. We also performed field potential recordings to measure the resulting field potentials and assess the effects of bath application of 50 μM MPP^+^. These recordings showed that the ratio of the two field potentials in WT and *Ask1*^−/−^ mice was not altered when using the paired-burst interval stimulation protocol with an inter-burst interval (IBI) of 10 or 40 s (see Supplementary Figures S2A,B). These electrophysiological methods were essential for controlling stimulation and measuring the resulting field potentials during the experiment.

### 2.6 Fluorescence imaging

**Figure 2:**
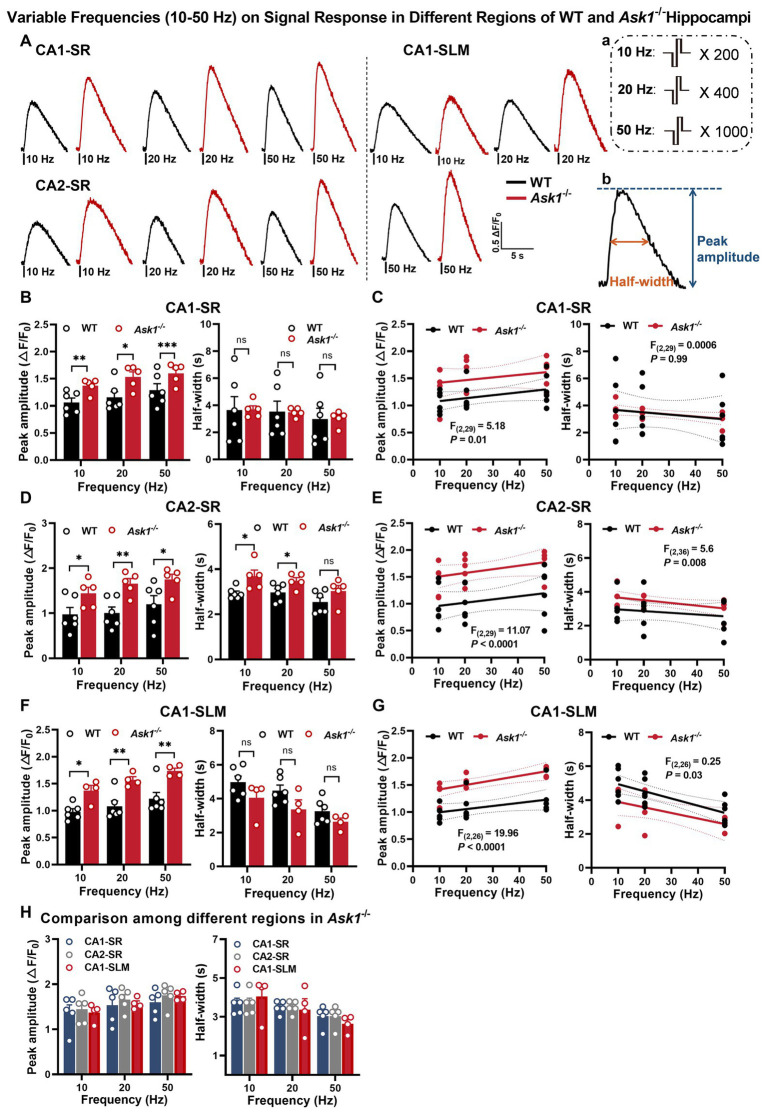
Enhanced stimulation and low-frequency-dependent dopamine release in the hippocampal region of *Ask1*^−/−^ mice. **(A)** The figure shows representative fluorescence traces of both WT and *Ask1*^−/−^ mice in response to different stimulation frequencies (ranging from 10 to 50 Hz) in the CA1-SR, CA2-SR, and CA1-SLM regions. Stimulation protocol (a) and the measurement of peak amplitude and half-width (b) were inserted. **(B,D,F)** Bar graphs present the peak values (left) and half-widths (right) of the fluorescence signals for different stimulation frequencies in CA1-SR **(B)**, CA2-SR **(D)**, and CA1-SLM **(F)** regions. ^*^p < 0.05, ^**^p < 0.01, ^****^p < 0.0001. **(C,E,G)** Scatter plots depict the individual data points, linear regression line, and confidence intervals of regression for peak ΔF/F_0_ values and half-width in CA1-SR **(C)**, CA2-SR **(E)**, and CA1-SLM **(G)** regions. Comparisons of regression are indicated. **(H)** Bar graphs provide a direct visual comparison of the peak fluorescence amplitude (on the left) and half-width values (on the right) among experiments conducted in the CA1-SR, CA2-SR, and CA1-SLM regions of *Ask1*^−/−^ mice. RM-TW-ANOVA: effect of regions: *p =* 0.53, *F*_(2, 33)_ = 0.64 (peak amplitude); *p =* 0.97, *F*_(2, 33)_ = 0.03 (half-width); Tukey’s multiple comparisons test: there were no significant differences among regions (the number of data points in the graphs corresponds to the number of slices from four mice). Comparison of regression fits between WT and *Ask1*^−/−^ mice is indicated [*F*_(DFn, DFd)_, *p*-value].

Following 2-h incubation periods, hippocampal slices were transferred to a recording chamber equipped with a 16× water-dipping objective (NA: 0.8, Nikon, Japan). To capture fluorescence changes, we sequentially activated different components. For illumination and excitation of the dLight dopamine sensor (filters: EX 480\40, BA 535\50), we utilized a TTL-controlled LED light source (X-Cite 120LED; Excelitas, Waltham, Massachusetts, USA). Images were then captured in time-lapse mode at a frequency of 33 Hz with an exposure time of 24 ms using a PCO.Edge 5.5 sCMOS camera (PCO AG, Kelheim, Germany). The activation of these components was controlled by the Clampex software, which also governed the stimulus isolator through the 5 V D/A outputs. Before capturing fluorescence changes, the LED light source was activated 1 s before the camera and 6 s before the stimulus patterns. To capture the fluorescence signal and reduce bleaching, the duration of illumination and recording was optimized after initial experiments. The fluorescence signal was measured from the brightest area adjacent to the stimulation electrode. For the time series analysis, we calculated ΔF/F_0_, where F_0_ represents the average intensity during the baseline period before electrical stimulation. Specifically, F_0_ was determined by averaging the intensity values from 2 s before stimulus onset to stimulus onset. ΔF is then calculated as the difference between F and F_0_. Pearson’s correlation coefficients (*r*) were calculated using GraphPad Prism 8.3.0 software, and linear regressions were compared across different experimental conditions. Peak amplitude and half-width were measured in the individual fluorescence curve using Clampfit 10.5 software. The peak amplitude is defined as the highest point of the curve when baseline values are subtracted, and the half-width (Dong et al., 2008) represents the time duration from the position where the signal reaches 50% of the peak amplitude during the rising phase to the point where a line, starting from the 50% peak amplitude position, intersects the decay phase of the signal (Figure 2B). The paired-burst ratios were normalized before drug application.

### 2.7 FFN 511 labeling of dopaminergic vesicles

To examine the presynaptic dynamics of dopaminergic vesicles, we utilized the fluorescent false neurotransmitter 511 (FFN 511, abcam, ab120331) to label these vesicles (Zhang et al., 2009). FFN 511 is a dye that incorporates itself into vesicles containing dopamine, enabling the monitoring of vesicle usage during dopamine release. Slices were incubated individually in loading solution (10 μM FFN 511 diluted in aCSF) for 30 min at room temperature (RT) following a 2-h incubation in aCSF at 32°C. To remove the extracellularly bound dye, the slice was subsequently transferred to 100 μM ADVASEP-7 (Sigma-Aldrich, A3723) aCSF for 30 min at RT. Subsequently, the FFN 511-loaded hippocampal slices were transferred to the recording chamber and subjected to imaging and analysis similar to the protocols used for dLight1.2 fluorescence imaging. The decrease in fluorescence signal corresponds to dopamine release.

### 2.8 RNA sequencing

Hippocampal slices from *Ask1*^−/−^ and *Ask1*^+/+^ mice were collected after incubation at 32°C for 2 h or in MPP^+^ after incubation at 32°C for one and a half hours and rapidly frozen in liquid nitrogen. The total RNA of these samples was isolated and used for RNA sequencing analysis. The cDNA library was constructed by Huada Gene Science and Technology Service Company (Shenzhen, China) on a DNBSEQ-T7. The sequencing data were filtered with SOAPnuke (Li et al., 2008), and differential expression analysis was performed using DEGseq (Wang S. H. et al., 2010) with a *Q* ≤ 0.05 (or FDR ≤0.001). The protein–protein interactions (PPIs) in the functional protein association network were constructed using the STRING database and visualized using Cytoscape, which is an open-source platform for complex networks. Only connections with a confidence score > 0.4 were selected, and unconnected genes were hidden. To explore potential relationships between protein interactions within this PPI network, the Cytoscape plugin ClusterONE (Nepusz et al., 2012) was used to detect subnetworks that met the following criteria: nodes >5, density >0.5, quality >0.5, and *p* < 0.05.

The SRA records will be accessible at: https://dataview.ncbi.nlm.nih.gov/object/PRJNA1045080.

### 2.9 Quantitative real-time polymerase chain reaction

Total RNA was extracted from hippocampal slices using the TaKaRa MiniBEST Universal RNA Extraction Kit (#9767, Takara, Kusatsu, Japan). The RNA was reverse transcribed into cDNA using the PrimeScript™ RT reagent Kit and gDNA Eraser (#RR047A, Takara). The cDNAs were then amplified using the QuantStudio 3 Real-Time PCR equipment with TB Green^®^ Premix Ex Taq™ II (#RR820A, Takara). The primers were mixed in, and the following PCR cycling parameters were used: 95°C for 1 min, followed by 40 cycles of 95°C for 30 s, 60°C for 40 s, and 72°C for 30 s. The data were exported from the system after normalization to GAPDH expression, and the 2^−ΔΔct^ calculation method was used for further evaluation. Supplementary Table S2 provides the primer sequences for all target genes.

### 2.10 Immunohistochemistry

For the immunohistochemistry procedure, mice were deeply anesthetized with 2.5% Avertin and then perfused with ice-cold normal saline followed by 4% paraformaldehyde after injection with dLight1.2. The whole brain was collected and placed in 4% paraformaldehyde overnight. Afterward, the brain was immersed in a 30% sucrose solution for 3 days and sliced into sections with a thickness of 25 μm using a cryostat (Leica 1900, Leica, Germany). These sections were then blocked with 10% goat serum, stained with a GFP antibody, and finally, the expression of dLight was detected using an Alexa Fluor 488-conjugated secondary antibody.

### 2.11 Western blotting

Hippocampal slices were collected in the same manner as for RNA sequencing. The protein concentrations were determined using a BCA Protein Assay Kit (Beyotime Institute of Biotechnology). Subsequently, the protein samples were denatured at 95°C and separated via 10% sodium dodecyl sulfate-polyacrylamide gel electrophoresis. After transferring the samples onto polyvinylidene fluoride membranes and blocking them with 5% non-fat milk, the membranes underwent overnight incubation at 4°C with primary antibodies. The membranes were then incubated with horseradish peroxidase-conjugated anti-mouse or anti-rabbit secondary antibodies for 2 h at room temperature. Finally, the immunosignals were detected using enhanced chemiluminescence (Pierce), and the resulting bands were visualized with the FluorChem E system (ProteinSimple, United States).

### 2.12 Statistical analysis

Statistical analysis was performed using GraphPad Prism 8 (GraphPad Software Inc.) and Clampfit (Molecular Devices, Sunnyvale, CA). All results are presented as mean ± SEM. The significance levels were set at ^*^p < 0.05, ^**^p < 0.01, ^***^p < 0.001, and ^****^p < 0.0001. Two-way analysis of variance (TW-ANOVA) with either repeated measurements (RM) or classical and multiple comparison tests was employed to determine statistical significance among different experimental conditions. Multiple slices were taken per mouse for the experiments, and the number of mice used is indicated in the figure legends.

## 3 Results

### 3.1 Temporal dynamic of fluorescence changes of dLight1.2 in response to electrical stimulation in stratum radiatum of the hippocampal CA1 region

**Figure 3:**
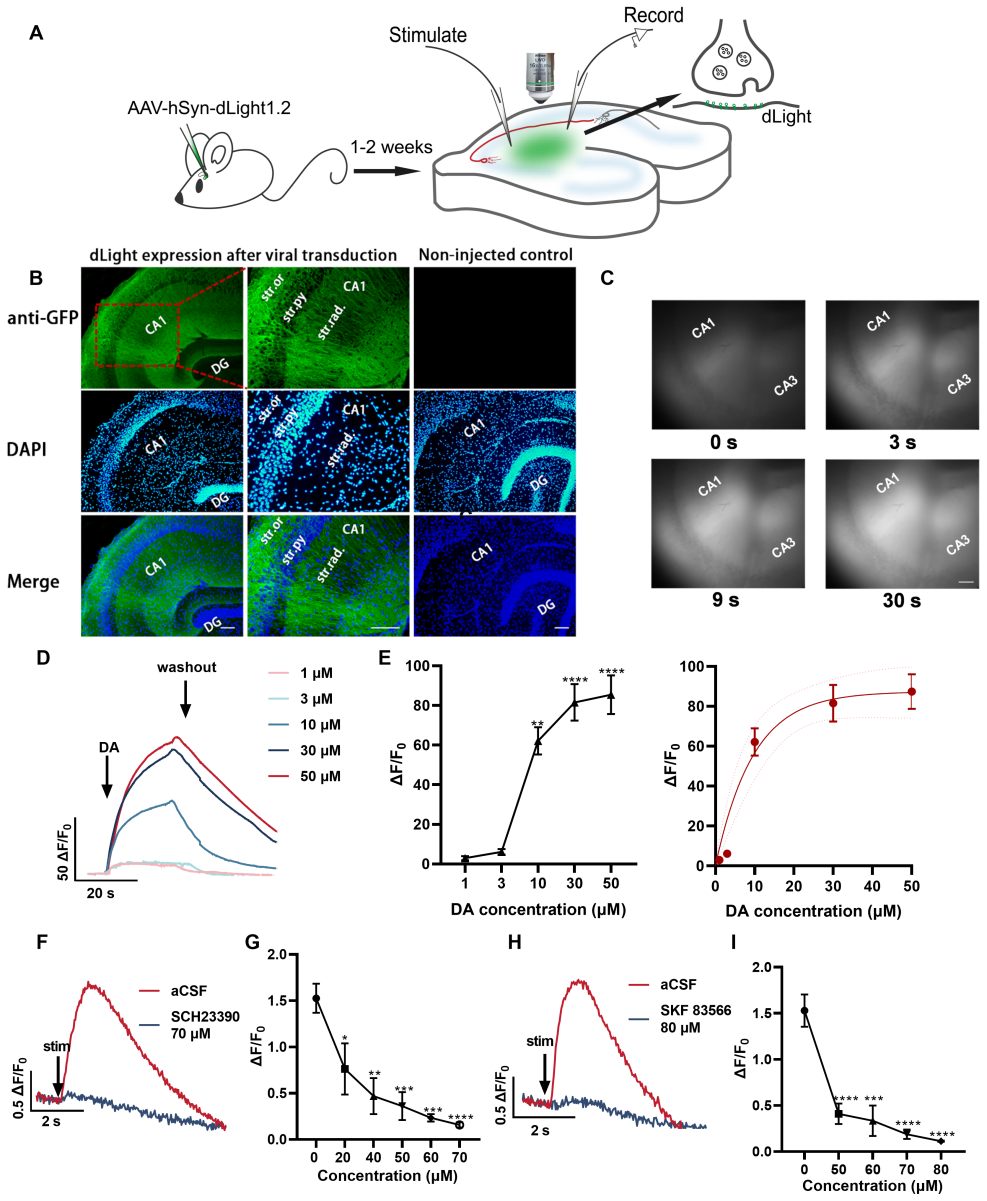
Unraveling the temporal dynamics of dopamine release in hippocampal slices using the fluorescent dopamine sensor dLight1.2. **(A)** Schemata illustrating the experimental approach for monitoring dopamine changes using the dLight fluorescent sensor in response to electrical stimulation. **(B)** Anti-GFP immunofluorescence images indicating the expression pattern of dLight1 following stereotaxic injection of AAV9-hSyn-dLight1.2 in the hippocampus. The images are merged with DAPI fluorescence images (bottom, blue). Higher magnification images are presented. Scale bar: 100 μm. Str. ori.: stratum oriens; Str. pyr.: stratum pyramidale; Str. rad.: stratum radiatum; DG: dentate gyrus. **(C)** Representative fluorescence images at different time points in the presence of dopamine (DA). Scale bars = 100 μm. **(D)** Averaged fluorescence traces in response to bath application of dopamine (1–50 μM). **(E)** Scatter plot demonstrating dopamine concentration-dependent fluorescence changes; statistical significance is indicated as ^**^p < 0.01, ^****^p < 0.0001 (compared to before dopamine application) and the fitted data curve on the right (five mice). **(F)** Representative fluorescence traces in response to 100 pulses at 100 Hz alone and the most significant response in the presence of SCH 23390 (70 μM, D1R antagonist). **(G)** Quantification of peak ΔF/F0 values at different SCH 23390 concentrations; ^*^p < 0.05, ^**^p < 0.01, ^***^p < 0.001, ^****^p < 0.0001 versus before application (five slices from four mice). **(H)** Fluorescence traces in aCSF and the most significant response in the presence of SKF 83566 (80 μM, D1R antagonist). **(I)** Plot showing the values for SKF 83566 experiments; ^***^p < 0.001, ^****^p < 0.0001 versus before application (five slices from four mice). Stim: time point of stimulation.

In this study, we utilized the genetically encoded fluorescent dopamine sensor, dLight1.2, to investigate the spatiotemporal release properties of dopamine in response to various stimulation patterns. This sensor has previously been used to characterize dopamine release in the striatum, nucleus accumbens, and cortex (Patriarchi et al., 2018). We injected adeno-associated virus encoding dLight1.2 (AAV9-hSynapsin1-dLight1.2) into the CA1 region of the intermediate area of the hippocampus (Figure 3A). Anti-GFP immunofluorescence indicates the widespread expression of dLight1.2 in the CA2 and CA1 regions of the hippocampus (Figure 3B). To verify the functionality of the sensor, dopamine was bath-applied. The dopamine bath applications resulted in a rapid increase (reaching maximum within 20 s), followed by a gradual decrease in fluorescence. The extent of fluorescence changes correlated with dopamine concentration (1–50 μM, Figures 3C–E). Additionally, we observed that stimulations with 100 pulses at 100 Hz induced a transient increase in fluorescence, which was blocked by D1R antagonists SCH 23390 (Figures 3F,G) and SKF 83566 (Figures 3H,I). These initial results confirm that the expression level of dLight1.2 in the hippocampal regions was sufficient to monitor dopamine release in response to brief burst stimulation using time-lapse fluorescence imaging. Although our measurements are qualitative in nature, dLight1.2 provides valuable insights into the dynamics of dopamine signaling. Considering the sensitivity of different dopamine detection methods, fast-scan cyclic voltammetry is known for its sensitivity, typically detecting dopamine at a limit of approximately 30–50 nM. In contrast, evoked responses in the dorsal striatum are typically in the higher range, approximately 1 μM (Patel and Rice, 2013). Comparatively, dLight1.2 has a lower sensitivity, with a Kd of 765 nM (Patriarchi et al., 2018). Despite its lower sensitivity, the dLight1.2 sensor captures the temporal and spatial characteristics of dopamine release in brain tissue, offering valuable insights into its dynamic behavior.

### 3.2 Comparison of pulse- and frequency dependency of dopamine release within hippocampal regions

**Figure 4:**
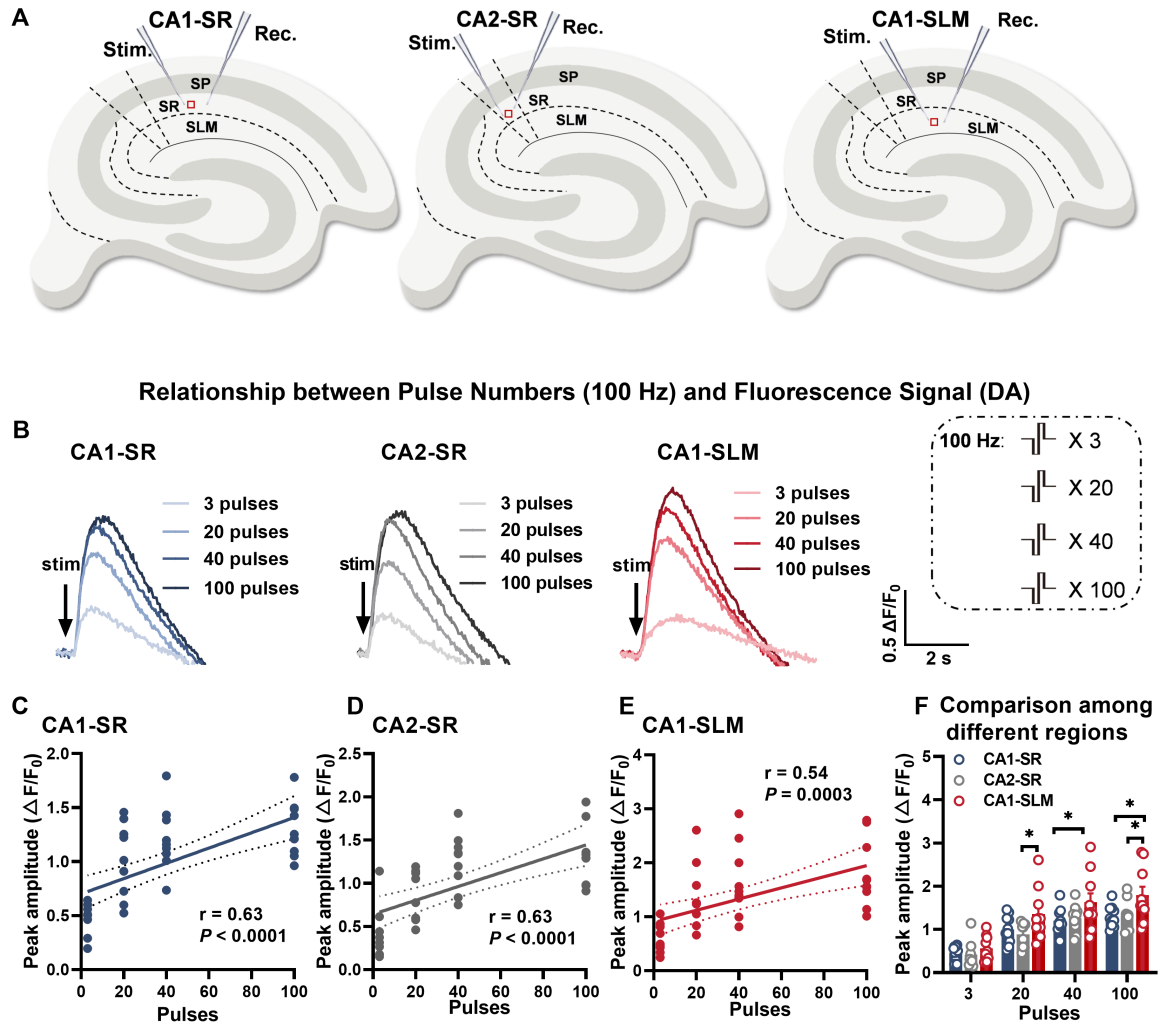
Evoked dopamine release depends on the number of stimuli and the hippocampal region. **(A)** Schematic diagrams illustrating the locations of stimulation and recording electrodes in different regions of the hippocampus, including the stratum radiatum of CA1 (CA1-SR), the stratum radiatum of CA2 (CA2-SR), and the stratum lacunosum-moleculare of CA1 (CA1-SLM). **(B)** Representative fluorescence traces demonstrating the response to a sequence of pulses (stimuli) at 100 Hz in the CA1-SR, CA2-SR, and CA1-SLM regions of the hippocampus. Stimulation protocol is inserted. **(C–E)** Scatter plots presenting ΔF/F0 values, linear regressions, and confidence intervals of regressions for peak values in the CA1-SR **(C)**, CA2-SR **(D)**, and CA1-SLM **(E)** regions. The Pearson’s correlation coefficient (*r*) and corresponding *p*-values are indicated within the plots. **(F)** Direct visual and statistical comparison of peak fluorescence amplitude among experiments in the CA1-SR, CA2-SR, and CA1-SLM regions. RM-TW-ANOVA: effect of regions: *p* = 0.0015, *F* (2, 36) = 7.856 (peak amplitude); *p* < 0.0001; Tukey’s multiple comparisons test: ^*^p < 0.05 (the number of data points in the graphs corresponds to the number of slices from four mice).

Dopaminergic fibers are distributed widely but in varying patterns throughout the hippocampus, originating from distinct brain regions (Takeuchi et al., 2016). The hippocampus receives dopaminergic input from various areas, including the ventral tegmental area (VTA), substantia nigra pars compacta (SNc), and locus coeruleus (LC). The VTA (Lisman and Grace, 2005) and SNc (Tsetsenis et al., 2021) are the primary sources of dopaminergic modulation in the hippocampus, primarily projecting to CA1, subiculum, and dentate gyrus. In contrast, the LC (Kempadoo et al., 2016; Takeuchi et al., 2016) provides more diffuse dopaminergic innervation throughout the hippocampus. This diverse input allows dopamine to modulate different aspects of hippocampal function in a subregion-specific manner. Consequently, there may be variations in innervation and dopamine release patterns across hippocampal subregions. The distinct origins and densities of dopaminergic fibers in each subregion, coupled with the unique arrangement of input–output fibers, could contribute to the heterogeneity of dopamine signaling. Given this diversity in hippocampal dopaminergic circuitry, our objective was to characterize the specific frequency-dependent profiles of dopamine release in the stratum radiatum of CA1 (CA1-SR), stratum radiatum of CA2 (CA2-SR), and stratum lacunosum-moleculare of CA1 (CA1-SLM) subregions (Figure 4A). By examining how dopamine release fluctuates in response to different activation frequencies, we aimed to gain insights into the modulation of hippocampal dopamine neurotransmission and analyze the variations in dopaminergic responsiveness to different stimuli across these unique subregions.

Considering that activity-dependent synaptic plasticity can be induced by various stimulation patterns that mimic intrinsic neuronal network activity, we employed a range of different stimulation patterns to induce dopamine release. To ensure comparability of the resulting dopamine release across different stimulations over time, we initially assessed whether photobleaching might affect the peak amplitudes when a sequence of identical stimulations (100 pulses at 100 Hz) was applied every 5 min. Regression analysis indicated that the degree of bleaching in these experiments did not have an impact on the peak amplitude or half-width over time (Supplementary Figures S3A–E). To achieve this, biphasic pulses with a phase duration of 0.2 ms were applied using different combinations of pulse numbers and frequencies (Figures 1A–F). Among these stimulation patterns, paired-burst stimulation was employed to provide a more detailed characterization of the dynamic changes in dopamine release recovery. The interval between stimulation trains was set at 5 min. We initially observed that, with stimulation patterns fixed at 100 Hz, there was a positive correlation between the number of stimuli at a fixed frequency and the increase in peak and half-width values. The representative fluorescence traces for the set of stimulations clearly displayed the rapid rise and decay of dopamine release induced by the train stimulation (Figure 4B). A comprehensive representation of electrode positions and resulting fluorescence changes in response to different pulse numbers and across different regions is presented in Supplementary Figure S4. The data demonstrated a moderate correlation between the number of stimuli and peak fluorescence in the CA1-SR region (Figure 4C, Pearson’s correlation coefficient (*r*) = 0.63), CA2-SR region (Figure 4D, *r* = 0.63), and CA1-SLM region (Figure 4E, *r* = 0.54). In summary, the ΔF/F_0_ peak values showed significant differences between the CA1-SLM region and other regions for a larger number of pulses at 100 Hz (Figure 4F).

Furthermore, in order to assess the potential impact of the initial fEPSP size, we examined the relationship between the linear regression slopes and the initial fEPSP values of the first stimulation protocol. Supplementary Figures S5B,C demonstrate that the fluctuations in the initial fEPSP size, which reflect the achieved level of stimulation, did not exhibit any significant correlation with the corresponding regression slope values (m) across the individual experiments (Supplementary Figures S5A–C).

We further investigated the contribution of stimulation frequency to dopamine release at a fixed number of 20 pulses. We found no significant correlation between different frequencies (100, 200, and 400 Hz) and peak ΔF/F_0_ (Supplementary Figures S6A–D). Moreover, no differences were observed among different hippocampal regions (Supplementary Figure S6E).

The theta-burst paradigm is designed to mimic two key features of the hippocampal network: burst-like afferent fiber activity and subsequent synaptic transmission resulting from complex spike patterns in adjacent hippocampal regions (DG, CA3) or remote brain areas. It also mimics the rhythmic modulation of excitability in these cells during the theta rhythm. This paradigm has been shown to effectively induce long-term potentiation (LTP) and has been extensively studied (Larson and Munkácsy, 2015). In our study, we used theta-burst-based stimulation and found no significant differences among the tested regions (Supplementary Figures S7E). Additionally, we did not observe any significant correlation between multiple theta bursts and either peak amplitude or half-width of dopamine release (Supplementary Figures S7A–E). In summary, our findings suggest that dopamine release in the hippocampal formation is driven by individual pulsatile stimuli rather than by overall frequency or theta-burst patterns.

### 3.3 Temporal dynamics of dopamine release in response to low-frequency and paired-burst stimulations

Synaptic potentiation is the strengthening of synaptic connections between neurons and is often associated with processes such as learning and memory. Interestingly, frequencies below 100 Hz have also been found to elicit long-term potentiation (LTP) (Dudek and Bear, 1992). In our study, we investigated the effects of various low stimulation frequencies (10–50 Hz) on dopamine release (Supplementary Figure S8). Surprisingly, we did not observe any correlation between peak ΔF/F_0_ and frequency in the CA1-SR (Supplementary Figure S8B), CA2-SR (Supplementary Figure S8C), and CA1-SLM (Supplementary Figure S8D) regions. However, the half-width of the dopamine release showed a moderate negative correlation with frequency in all three areas, with significant *p*-values found in CA1-SLM (*p* = 0.009), CA1-SR (*p* = 0.02), and CA2-SR (*p* = 0.03) (Supplementary Figure S8). Furthermore, when comparing the peak amplitude and half-width among different regions, no significant differences were observed (Supplementary Figure S8E).

**Figure 5:**
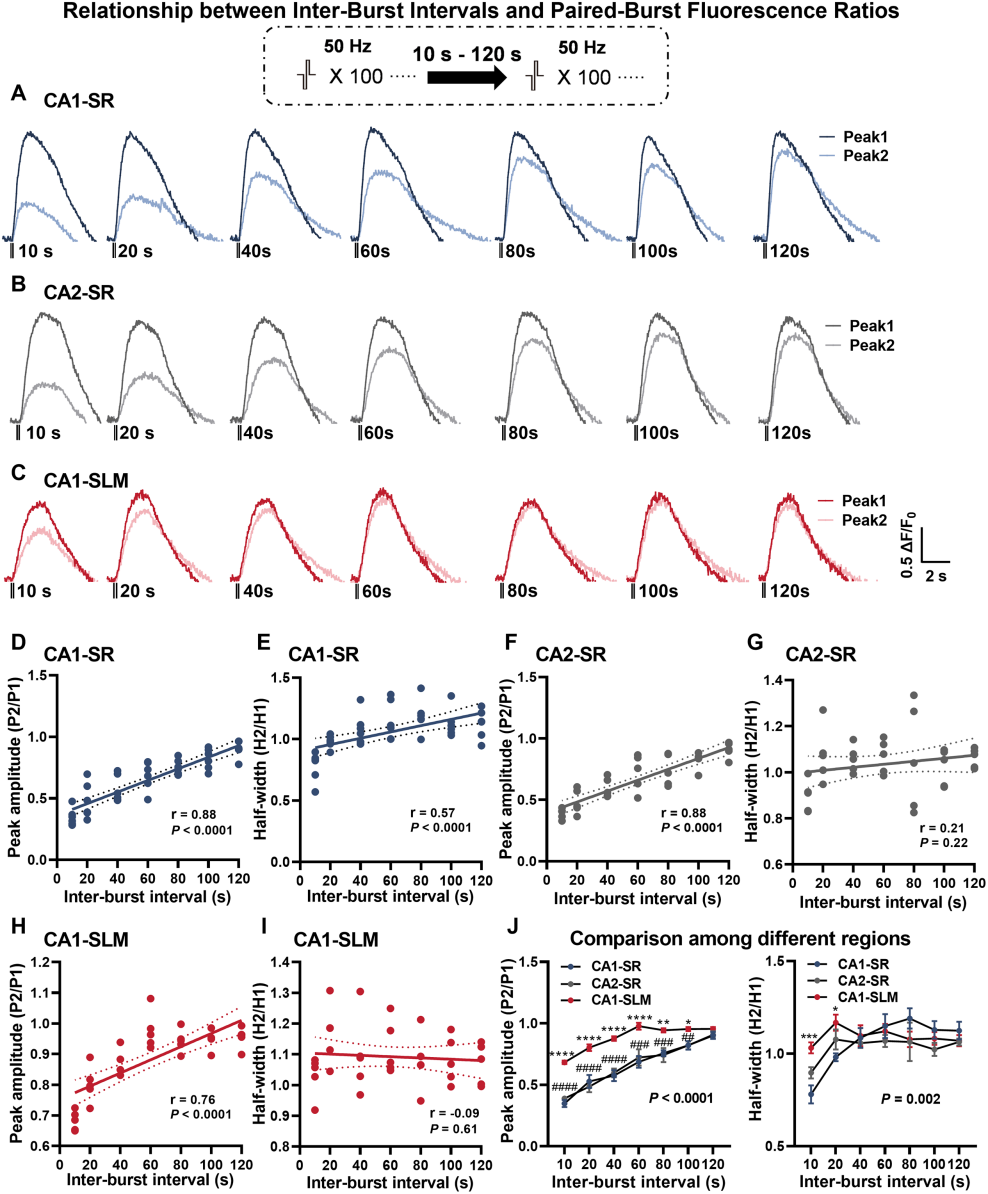
Dependency of dopamine release on different paired-burst intervals. **(A–C)** Average fluorescence traces in response to paired-burst stimulation with inter-burst intervals ranging from 10 to 120 s within the specified hippocampal regions. Stimulation protocol was inserted. **(D–I)** Scatter plots present individual data points with linear regression lines and confidence intervals for peak ΔF/F_0_ and half-width values. The Pearson’s correlation coefficient (r) and corresponding *p*-values are indicated in each correlation plot. **(J)** The summary of mean data illustrates peak ΔF/F_0_ values (left) and half-width values (right) per paired-burst stimulation for different regions. RM-TW-ANOVA, effect of regions: *p* < 0.0001, *F*_(2, 13)_ = 34.02 (peak amplitude); *p* = 0.10, *F*_(2, 91)_ = 2.32 (half-width); Tukey’s multiple comparisons test: CA1-SR and CA1-SLM: ^*^p < 0.05, ^**^p < 0.01, ^***^p < 0.001, ^****^p < 0.0001; CA1-SR and CA2-SR: ^##^*p* < 0.01, ^###^*p* < 0.001, ^####^*p* < 0.0001 (the number of data points in the graphs corresponds to the number of slices from four mice).

To learn more about the time course of dopamine release recovery after stimulation-dependent depletion, we investigated dopamine release evoked by a protocol that consisted of two bursts with 100 pulses at 50 Hz with inter-burst intervals (IBI) of 10–120 s in CA1-SR (Figure 5A), CA2-SR (Figure 5B), and CA1-SLM (Figure 5C) regions. In these regions, we evaluated the ratio of dopamine release evoked by the second stimulation to that of the first stimulation. As the inter-burst interval (IBI) increased, the peak amplitude of the second dopamine release event approached that of the first stimulus, reaching full recovery within 120 s in CA1-SR and CA2-SR and 60 s in CA1-SLM. Notably, the observed paired-pulse recovery rate for dopamine release is slower than the recovery rate observed for glutamate release (Szerb and O’Regan, 1985).

The slow recovery of secondary dopamine release observed in the hippocampus is consistent with previous findings in the striatum (Kennedy et al., 1992; Phillips et al., 2002). Peak amplitude recovery exhibited a highly significant positive correlation with increasing IBIs in all regions examined, including CA1-SR (Figure 5D, *p* < 0.0001), CA2-SR (Figure 5F, *p* < 0.0001), and CA1-SLM (Figure 5H, *p* < 0.0001). Notably, the recovery of the second stimulus in CA1-SLM was more pronounced compared to the CA1-SR and CA2-SR regions. In terms of half-width, only the CA1-SR region exhibited a significant correlation with different IBIs (Figure 5E, *p* < 0.0001), while no such correlation was found in the CA2-SR (Figure 5G) and CA1-SLM regions (Figure 5I). Comparison of paired-burst fluorescence peak ratios across the different regions revealed that the CA1-SLM showed significantly less paired-burst inhibition for all IBIs in comparison with CA1-SR and CA2-SR (Figure 5J). However, in terms of half-width ratio, the CA1-SLM was only significantly different from CA1-SR and CA2-SR at 10- and 20-s IBIs (Figure 5J).

### 3.4 *Ask1* deficiency alters dopamine release kinetics compared to wild-type mice

**Figure 6:**
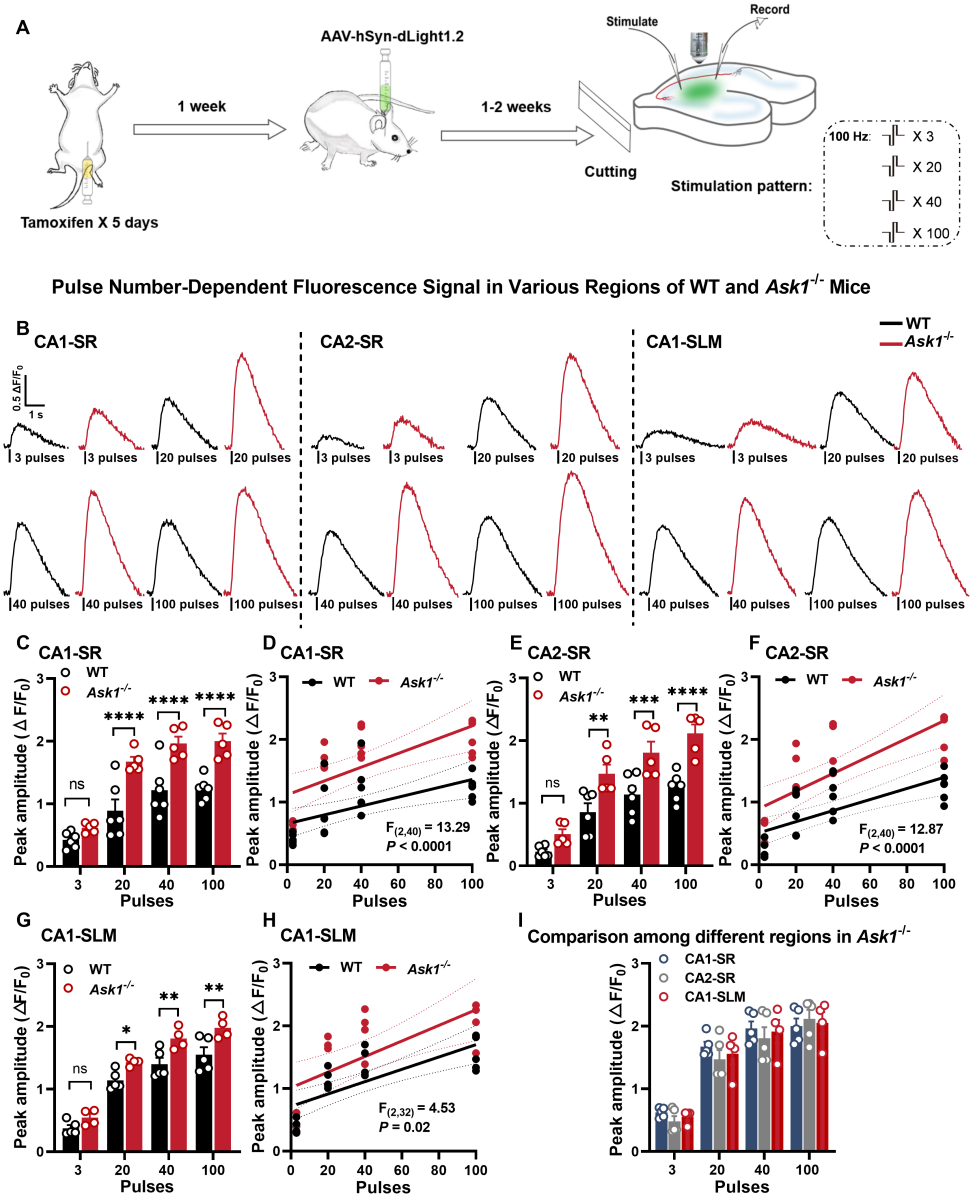
Enhanced dopamine release in *Ask1*^−/−^ mice with 100 Hz stimulations in the CA1-SR and CA2-SR regions. **(A)** Experimental design illustrating the setup for *Ask1*^−/−^ mice. **(B)** Representative fluorescence traces acquired in hippocampal slices from littermate wildtype (WT) (black traces) and *Ask1*^−/−^ (red traces) mice for different numbers of pulses at 100 Hz in the CA1-SR, CA2-SR, and CA1-SLM regions. Stimulation protocol was inserted. **(C,E,G)** Bar graphs show individual values, mean, and SEM for fluorescence peak values obtained from experiments conducted in the CA1-SR **(C)**, CA2-SR **(E)**, and CA1-SLM **(G)** regions. ^*^p < 0.05, ^**^p < 0.01, ^***^p < 0.001, ^****^p < 0.0001. **(D,F,H)** Scatter plots depicting individual data points, linear regression line, and confidence interval of regression for peak ΔF/F_0_ values in the CA1-SR **(D)**, CA2-SR **(F)**, and CA1-SLM **(H)** regions. Comparison of regression fits between WT and *Ask1*^−/−^ mice is indicated [F_(DFn, DFd)_, *p*-value]. **(I)** Direct visual comparison of the peak fluorescence amplitude among experiments in the CA1-SR, CA2-SR, and CA1-SLM regions of *Ask1*^−/−^ mice. The results demonstrate no significant differences among regions (RM-TW-ANOVA), effect of regions: *p* = 0.54, *F*_(2, 44)_ = 0.61 (peak amplitude); Tukey’s multiple comparisons test: no significant differences among regions (the number of data points in the graphs corresponds to the number of slices from four mice).

In this study, we investigated the response of dopamine release in *Ask1*-deficient mice to different stimulation protocols (Figure 6A). Similar to littermate wild-type (WT) mice, dopamine release in *Ask1*-deficient mice increased with an increasing number of stimuli in the CA1-SR (Figure 6C), CA2-SR (Figure 6E), and CA1-SLM regions (Figure 6G), and representative fluorescence traces were shown in Figure 6B. Specifically, the peak value of 20, 40, and 100 stimuli was significantly higher in *Ask1*^−/−^ than in WT in CA1-SR (Figure 6C), CA2-SR (Figure 6E), and CA1-SLM (Figure 6G). Thus, there was a strong correlation between the number of stimuli (3, 20, 40, and 100) and peak fluorescence in these three areas. However, the regression fit revealed significant differences between the regressions of WT and *Ask1*^−/−^ for each region, as shown in the graphs for CA1-SR (Figure 6D, *p* < 0.0001), CA2-SR (Figure 6F, *p* < 0.0001), and CA1-SLM (Figure 6H, *p* = 0.02). Overall, the dependencies of peak fluorescence on pulse numbers at 100 Hz were not different among different regions in *Ask1*^−/−^ (Figure 6I). In a separate set of experiments, we observed increases in peak dopamine release with frequencies of 10, 20, and 50 Hz in all regions (Figures 2B,D,F). However, when comparing the half-width, only CA2-SR showed a significant difference in half-width at 10 and 20 Hz between WT and *Ask1*^−/−^ (Figure 2D). These dependencies are also evident in the non-linear regression comparison between WT and *Ask1*^−/−^, where the statistical analysis revealed a significant difference in peak amplitude to frequency for all tested regions (Figures 2C,E,G). A similar analysis of the half-width frequency dependency revealed significant differences in the regressions between WT and *Ask1*^−/−^ for CA2-SR and CA1-SLM (Figures 2E,G). In terms of differences in peak and half-width values among different regions for *Ask1*^−/−^ alone, we did not detect any significant differences (Figure 2H).

**Figure 7:**
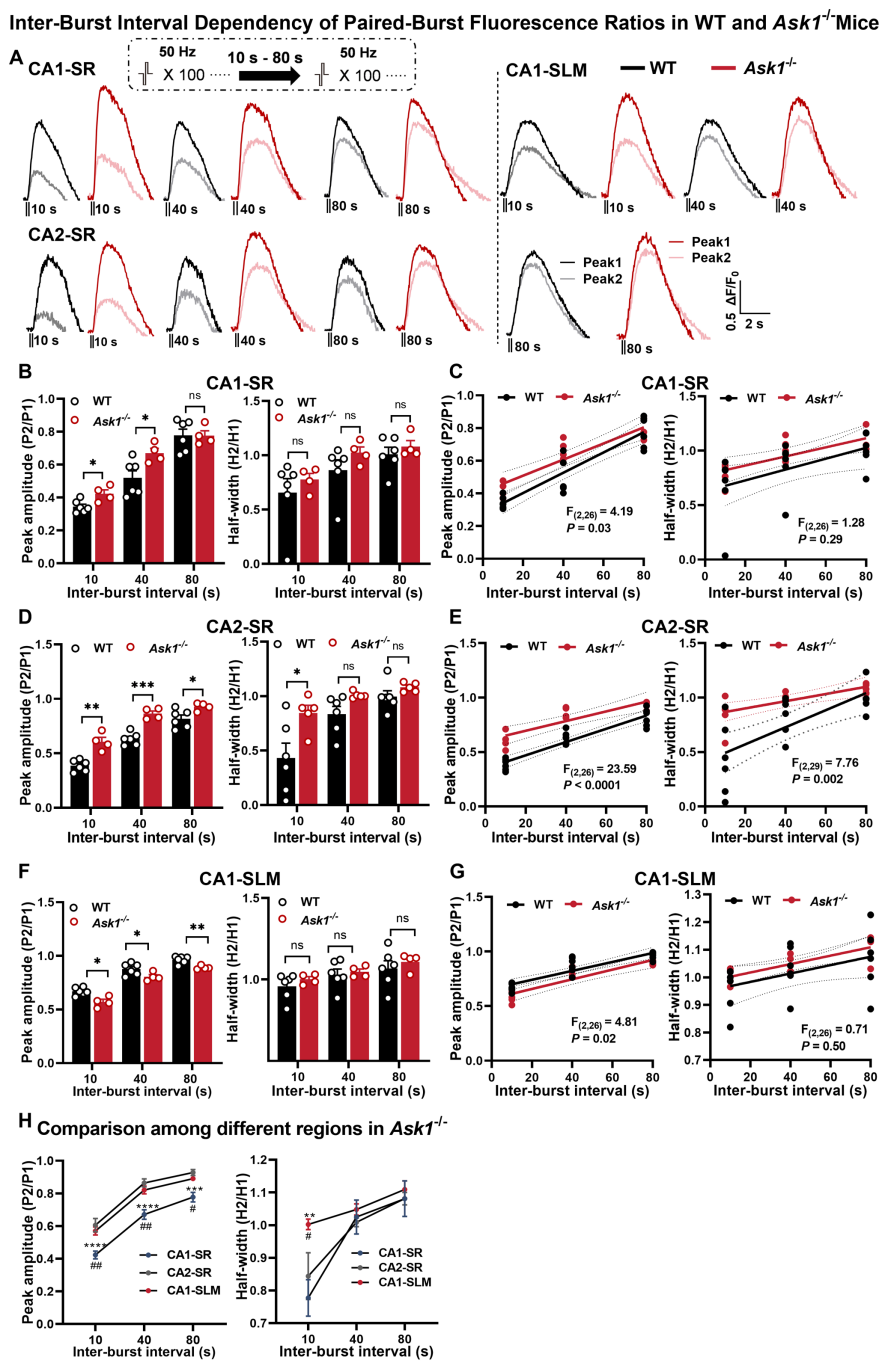
Comparison of fluorescence responses to paired-burst stimulation in WT and *Ask1*^−/−^ mice in the CA1-SR, CA2-SR, and CA1-SLM regions. **(A)** The panel illustrates representative pairs of fluorescence traces (peak 1 and peak 2) in response to paired-burst stimulation with inter-burst intervals between 10 and 80 s in the CA1-SR, CA2-SR, and CA1-SLM regions. Stimulation protocol was inserted. **(B,D,F)** Bar graphs present the peak (on the left) and half-width (on the right) values for WT (black) and *Ask1*^−/−^ (red) mice at different inter-burst intervals in CA1-SR **(B)**, CA2-SR **(D)**, and CA1-SLM **(F)** regions, ^*^p < 0.05, ^**^p < 0.01, ^***^p < 0.001, ^****^p < 0.0001. **(C,E,G)** Scatter plots display individual data points, linear regression lines, and confidence intervals of regression for the ratios of the △F/F_0_ values of peak 2 divided by peak 1 and the ratio of the two half-width values in CA1-SR **(C)**, CA2-SR **(E)**, and CA1-SLM **(G)** regions. Comparison of regression fits between WT and *Ask1*^−/−^ are indicated. **(H)** Comparison of the peak fluorescence amplitude (on the left) and half-width values (on the right) among experiments in the CA1-SR, CA2-SR, and CA1-SLM regions of *Ask1*^−/−^. RM-TW-ANOVA: effect of regions: *p* < 0.001, *F*_(2, 27)_ = 37.7 (peak amplitude); *p* = 0.034, *F*_(2, 30)_ = 3.782 (half-width); Tukey’s multiple comparisons test: CA1-SR and CA2-SR: ^**^p < 0.01, ^***^p < 0.001, ^****^p < 0.0001; CA1-SR and CA1-SLM: ^#^*p* < 0.05, ^##^*p* < 0.01 (the number of data points in the graphs corresponds to the number of slices from four mice). Comparison of regression fits between WT and *Ask1*^−/−^ mice is indicated [*F*_(DFn, DFd)_, *p*-value].

In the next step, we investigated whether *Ask1* deficiency affected the recovery of dopamine release using the paired-burst stimulation protocol with inter-burst intervals of 10, 40, and 80 s in comparison with WT mice (Figure 7). Our results indicated that *Ask1* deficiency significantly increased the peak amplitude ratio in the CA1-SR at 10- and 40-s intervals but not at 80 s, which reflects an enhanced recovery of dopamine release (Figure 7B). However, peak ratio values between WT and *Ask1* mice were higher at all IBS in the CA2-SR (Figure 7D), but significantly lower at all IBS in the CA1-SLM (Figure 7F). The different dependencies of fluorescence ratio to IBI were further supported by the comparison of regressions between WT and *Ask1*^−/−^ mice (Figures 7C,E,G). Regarding the decay time, characterized by the half-width, we did not detect any differences between WT and *Ask1*^−/−^ in most instances, except for the CA2-SR at 10-s IBI (Figure 7D). A significant difference in the half-width to IBI regressions between WT and *Ask1*^−/−^ was also observed. When comparing different regions in *Ask1*^−/−^ alone, we detected significantly lower peak ratios between CA1-SR and other regions for all IBIs, but not in terms of half-width. Regarding half-width, a higher value was only observed at 10-s IBI for CA1-SLM (Figure 7H).

### 3.5 *Ask1* deficiency attenuates the impairment of dopamine release recovery induced by MPP^+^

**Figure 8:**
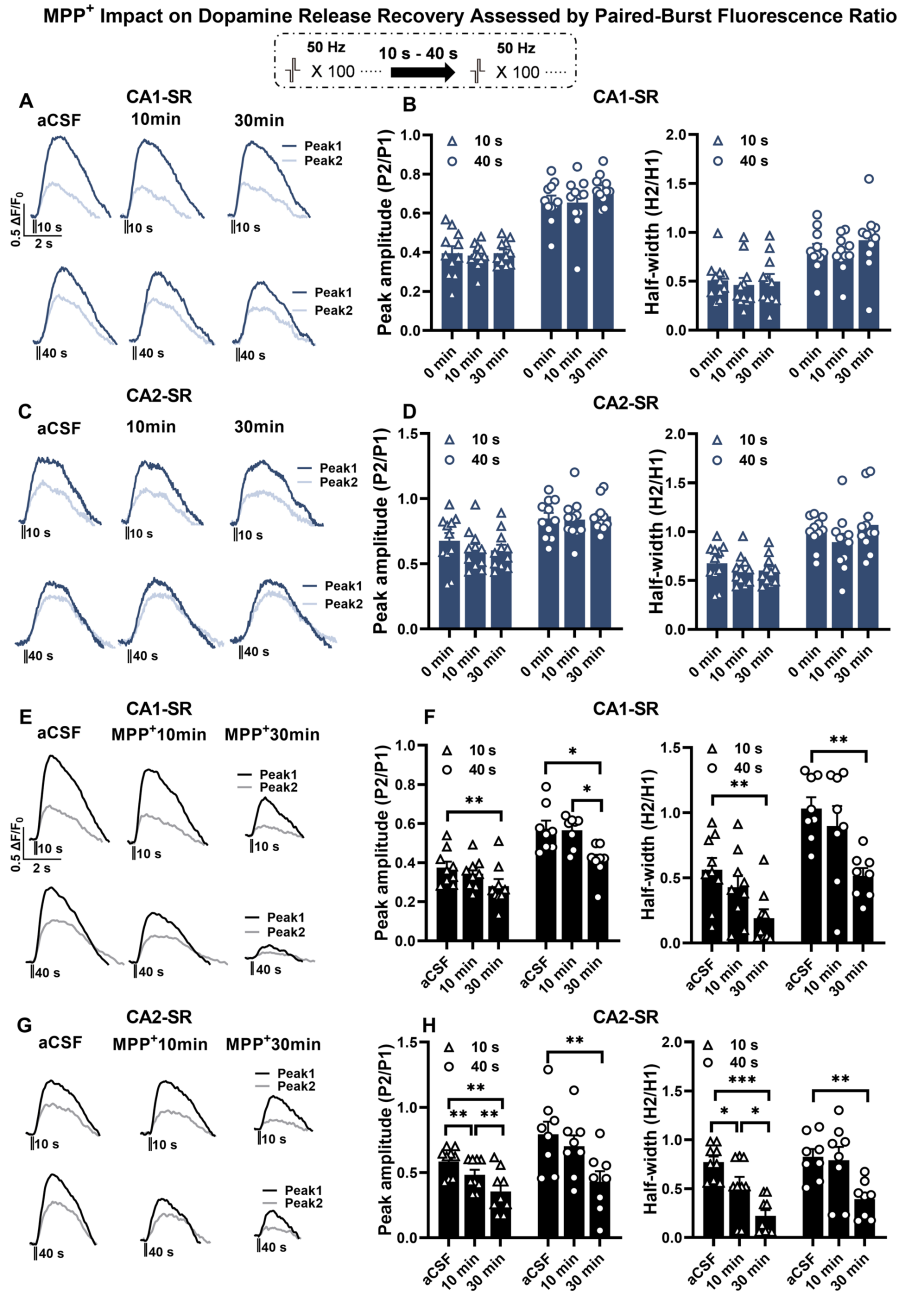
The effect of MPP^+^ on dopamine release recovery in paired-burst stimulation in the CA1-SR and CA2-SR regions. **(A,C)** Representative fluorescence traces of paired-burst stimulation with an inter-burst interval of 10 s or 40 s in the absence of MPP^+^ indicate the response to the first pulse (dark colors) and the second pulse (faded colors). Stimulation protocol was inserted. **(B–D)** The resulting mean ratios of the first and second peak amplitude △F/F_0_ (P2/P1) and half-width (H2/H1) under aCFS conditions followed by a 10- and 30-min incubation in CA1-SR **(B)** and CA2-SR **(D)** are presented. **(E,G)** Representative fluorescence traces of paired-burst stimulation with an inter-burst interval of 10 s or 40 s in CA1-SR **(E)** and CA2-SR **(G)** regions, indicating the response to the first stimuli (dark colors) and the second stimuli (faded colors). **(F,H)** Mean ratios of the first and second peak amplitude ΔF/F_0_ (P2/P1) and half-width (H2/H1) under aCSF conditions followed by a 10- and 30-min incubation with MPP^+^ in CA1-SR **(F)** and CA2-SR **(H)** (the number of data points in the graphs corresponds to the number of slices from four mice).

Next, we investigated the impact of bath-applied MPP^+^ on dopamine release. Initial experiments indicated that repeated stimulation and measurement of peak amplitude ratio and half-width ratio remained stable over 30 min (Figures 8A–D). Regarding the bath application of MPP^+^, our findings indicate that acute application of MPP^+^ (50 μM, 30 min) to hippocampal slices reduced the recovery of dopamine release in response to paired-burst stimulations with 10- and 40-s IBIs (Figures 8E–H). Specifically, exposure to MPP^+^ for 30 min resulted in a significant reduction in the fluorescence peak ratio and half-width ratio in the CA1-SR (Figures 8E,F) and CA2-SR (Figures 8G,H) of the hippocampus compared with the measurements taken before drug application (aCSF). The effect of MPP^+^ in the CA2-SR region was significantly different already after 10 min of bath application, while similar significance levels for the CA1-SR were only detected after 30 min.

**Figure 9:**
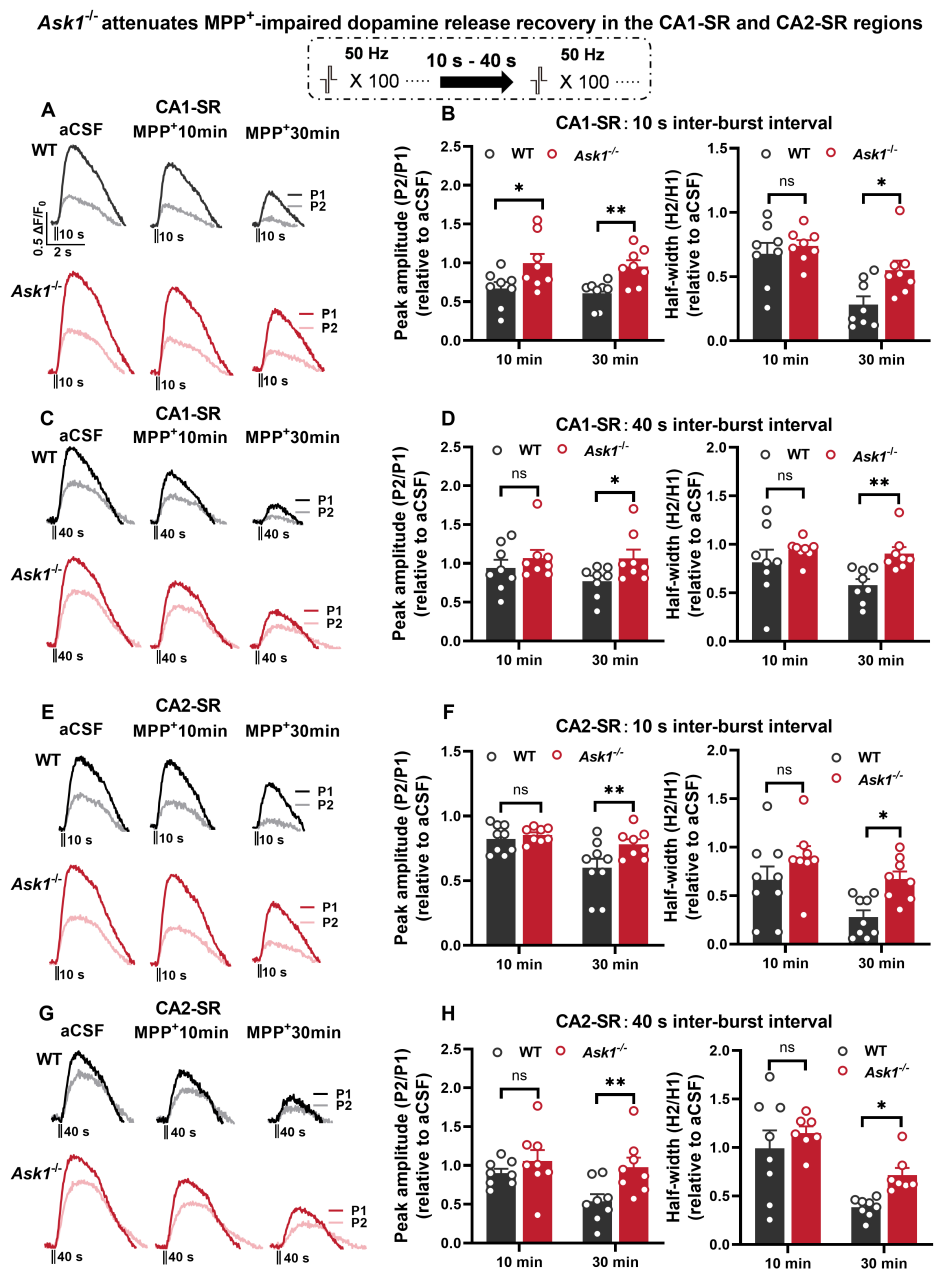
*Ask1* knockdown attenuates MPP^+^-impaired dopamine release recovery in the CA1-SR and CA2-SR regions. **(A,C)** Representative fluorescence traces displaying the response to paired-burst stimulation with 10-s **(A)** and 40-s **(C)** inter-burst intervals between WT and *Ask1*^−/−^ mice in the CA1-SR region. Stimulation protocol was inserted. **(B,D)** The graphs present data that have been normalized to the values before drug application (relative to aCSF measurements). The comparison of the fluorescence peak value ratio (P2/P1; left) and half-width ratio (H2/H1; right) between WT and *Ask1*^−/−^ in hippocampal CA1-SR was carried out in the presence of MPP^+^. **(E,G)** Representative fluorescence traces with or without MPP^+^ in response to paired-burst stimulation with 10-s **(E)** and 40-s **(G)** inter-burst intervals in the CA2-SR region. **(F,H)** The graphs present the ratios as normalized to the before drug application values (relative to aCSF). The comparison of the P2/P1 (left) and H2/H1 (right) values with 10-s **(F)** and 40-s **(H)** inter-burst intervals between WT and *Ask1*^−/−^ in the hippocampal CA2-SR was carried out in the presence of MPP^+^. *T*-tests were used for statistical analysis, and the results are denoted by ^*^p < 0.05 and ^**^p < 0.01 (the number of data points in the graphs corresponds to the number of slices from four mice).

We next proceeded to examine whether *Ask1* deficiency attenuates the MPP^+^ effect on paired-burst stimulation-evoked dopamine release. To be able to compare experiments more precisely from different slices, we normalize the peak ratio and half-width ratios to those before drug application (Figure 9). The results showed that the normalized peak ratio between WT and *Ask1*^−/−^ mice was significantly higher in *Ask1*^−/−^ mice at 10 min of MPP^+^ bath application in the CA1-SR for the 10-s IBI and at 30 min for the 40-s IBI. Regarding the half-width, a significant increase in *Ask1*^−/−^ mice compared with WT was observed 30 min after drug application (Figures 9A–D). In the CA2-SR region, similar dependencies were detected, except that all parameters were significantly higher at 30 min of drug bath application (Figures 9E–H).

**Figure 10:**
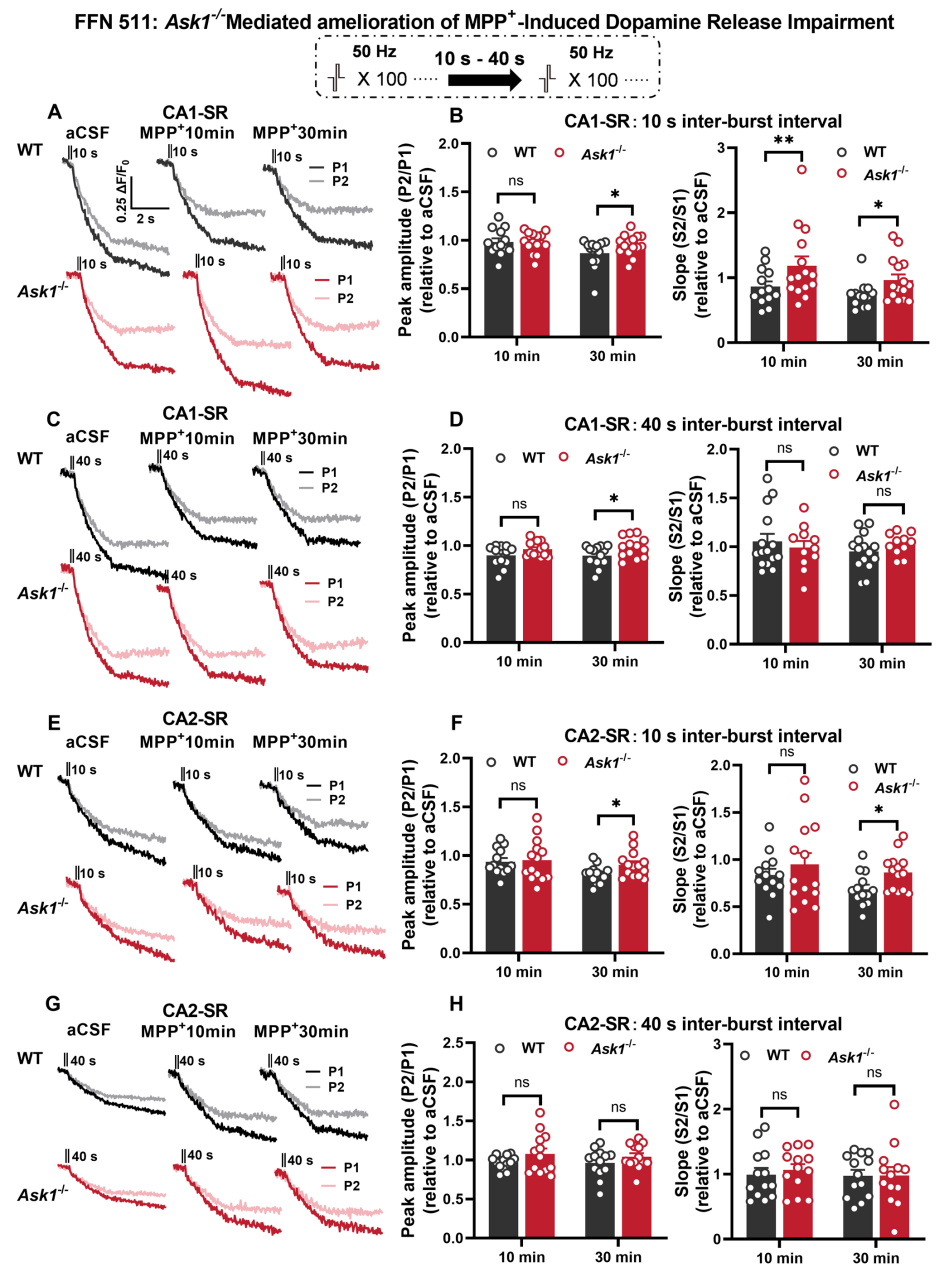
*Ask1* deficiency rescues the decrease in repeated vesicular dopamine release induced by MPP^+^ in the CA1-SR and CA2-SR regions. **(A,C)** Destaining curves of FFN 511 fluorescence were obtained in response to paired-burst stimulation with inter-burst intervals of 10 s or 40 s in the CA1-SR region before and after the application of MPP^+^. The stimulation protocol is inserted. **(B,D)** The comparison of the P2/P1 (left) peak ratios and S2/S1 (right) slope ratios was conducted for (B) 10-s and (D) 40-s inter-burst intervals, 10 and 30 min after the application of MPP^+^ in the CA1-SR region, and normalized to before drug application (aCSF). **(E,G)** Representative fluorescence traces with or without MPP^+^ in response to paired-burst stimulation with 10-s (E) and 40-s (G) inter-burst intervals were recorded. **(F,H)** The comparison of the P2/P1 (right) and S2/S1 (left) responses to paired-burst stimulation with 10- and 40-s inter-burst intervals in MPP^+^ between WT and *Ask1*^−/−^ in the hippocampal CA2-SR region was conducted. *T*-tests were used for statistical analysis, and the results are denoted by ^*^p < 0.05 and ^**^p < 0.01. The number of data points in the graphs corresponds to the number of slices from four mice.

As mentioned earlier, FFN 511 is incorporated into the dopaminergic vesicles, enabling the monitoring of vesicle utilization during dopamine release by measuring a reduction in fluorescence. This approach was used to investigate the effects of stimulation and MPP^+^ on vesicular dopamine release (Figure 10). After a 30-min MPP^+^ bath application, the normalized peak ratio for the 10- and 40-s IBIs showed a significant increase in *Ask1*^−/−^ compared with WT in the CA1-SR region (Figures 10A–D). When analyzing the decay slope as a second parameter, similar differences were observed between WT and *Ask1*^−/−^, although the slope remained non-significant for the 40-s IBI. In the CA2-SR region, significance was only found after a 30-min application, specifically for the normalized peak and slope ratio at the 10-s IBI (Figures 10E–H). Overall, the data suggest that *Ask1* deficiency attenuates the impairment of vesicular dopamine release caused by MPP^+^, resulting in a greater peak ratio at the 10-s IBI in both regions.

### 3.6 *Ask1* deficiency attenuates MPP^+^-induced dopamine impairment by regulating *Slc5a7* gene expression

**Figure 11:**
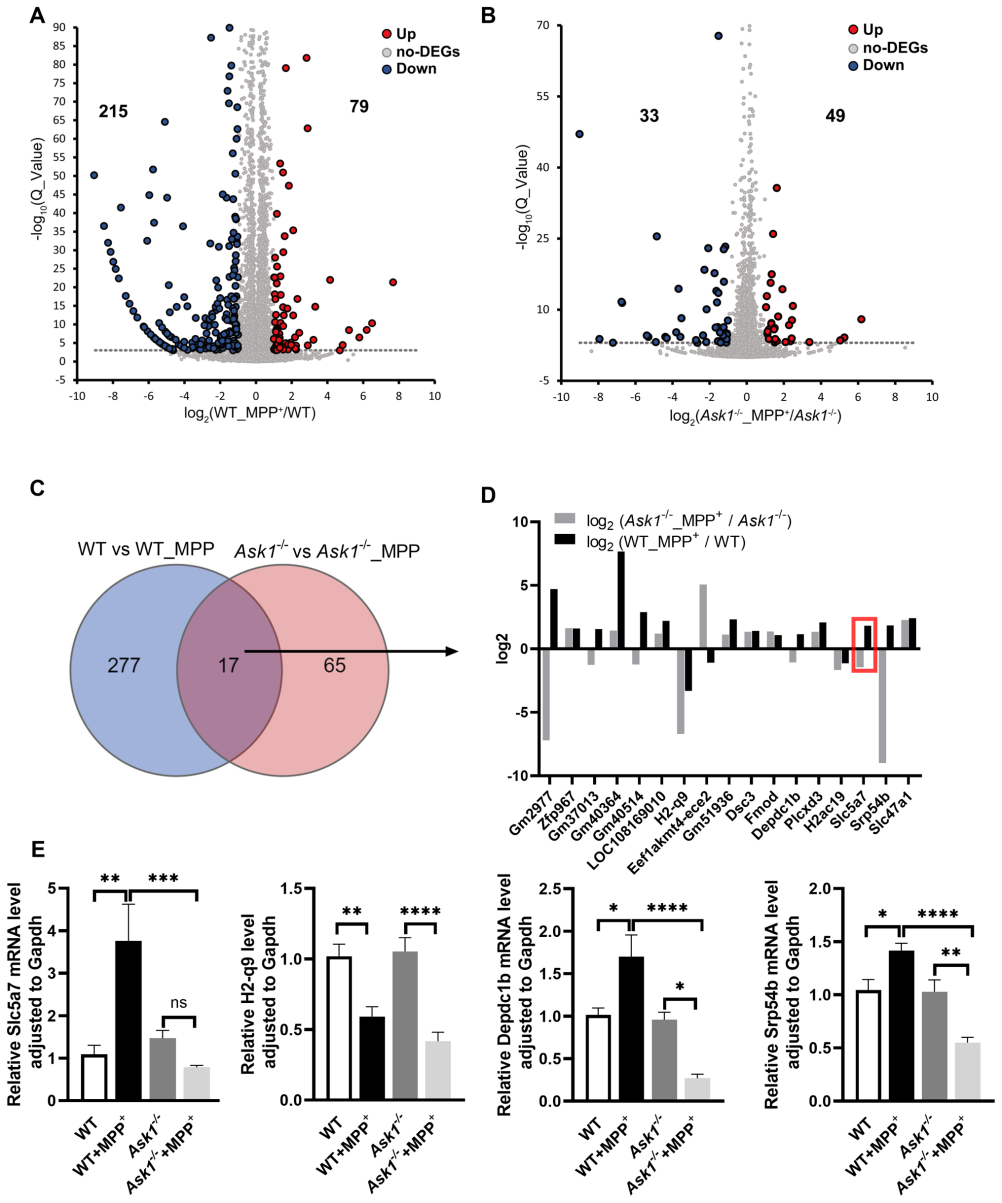
Partially overlapping gene expression pattern induced by MPP^+^ in WT and *Ask1*^−/−^ mice. **(A)** The volcano plot displays the differentially expressed genes (DEGs) identified in WT mice by comparing a 30-min application of MPP^+^ versus no drug application. **(B)** This volcano plot illustrates the distribution of up- (red) and downregulated (blue) DEGs in *Ask1*^−/−^-deficient mice in response to MPP^+^. The thresholds used to identify DEGs were fold change >|1| and *Q* value <0.001. **(C)** The Venn diagram highlights the overlap of DEGs in the response to MPP^+^ between WT (blue) and *Ask1*^−/−^ (red). **(D)** The log2 values and the names of the overlapping DEGs for the two comparisons (WT vs. WT_MPP^+^: gray; *Ask1*^−/−^ vs. *Ask1*^−/−^ + MPP^+^: black) are shown. For some of the overlapping DEGs, the directions of gene expression were opposite (three mice). In the red box is the *Slc5a7* gene. **(E)** The bar graph summarizes the qPCR values of the indicated genes across different genotypes and experimental conditions. Significant differences between experimental conditions are indicated by brackets and asterisks: One-way ANOVA and *post hoc* with multiple comparison correction (Sidak): ^*^p < 0.05, ^**^p < 0.01, ^***^p < 0.001, ^****^p < 0.0001 (six mice).

To investigate the potential regulation of gene expression by *Ask1*^−/−^ on a genome-wide level under MPP^+^ incubation conditions, we performed RNA sequencing analysis on hippocampal slices from both WT and *Ask1*^−/−^ mice. Figure 11 illustrates the differentially expressed genes (DEGs) following a 30-min incubation with MPP^+^ in WT mice (Figure 11A) and *Ask1*^−/−^ mice (Figure 11B). The Venn diagram (Figure 11C) shows the comparison of DEGs between *Ask1*^−/−^ + MPP^+^ versus *Ask1*^−/−^ and WT + MPP^+^ versus WT, revealing 17 overlapping DEGs. Notably, the log_2_ fold changes of these 17 DEGs displayed similar or opposite directions of gene expression levels in response to MPP^+^ in WT and *Ask1*^−/−^ mice (Figure 11D). Among the overlapping genes, *Srp54b* (signal recognition particle 54B) was identified, which is predicted to have 7S RNA-binding activity, endoplasmic reticulum signal peptide-binding activity, and guanyl ribonucleotide-binding activity. Another gene in this list is *H2-q9* (histocompatibility 2, Q region locus 9), which is involved in peptide antigen-binding activity. *Depdc1b* (DEP domain-containing 1B) is another overlapping gene that exhibits GTPase activator activity. Additionally, *Slc5a7* (solute carrier family 5 member 7) is among these 17 genes. *Slc5a7* encodes a high-affinity sodium/chloride ion-dependent transporter that mediates choline transport for acetylcholine (Ach) synthesis.

To confirm the relative expression levels of these four genes in different treatments and mice, we conducted qRT-PCR analysis (Figure 11E). While the expression levels of *Slc5a7* were comparable between WT and *Ask1*^−/−^ mice, there was a significant upregulation of *Slc5a7* in response to MPP^+^ treatment observed specifically in WT mice. Thus, the deficiency of *Ask1* prevents the induction of *Slc5a7* expression by MPP^+^ and even shows a tendency toward reduced *Slc5a7* expression compared to the drug-free samples (Figure 11E, left panel). Additionally, the expression level of *Slc5a7* is significantly different between the *Ask1*^−/−^ + MPP^+^ and WT + MPP^+^ groups. Overall, the qRT-PCR data validate the findings of the RNA sequencing analysis, confirming a lack of increased *Slc5a7* expression in response to MPP^+^ in *Ask1*^−/−^ mice. Furthermore, the qRT-PCR analysis of *H2-q9*, *Depcdc1b*, and *Srp54b* demonstrated changes in expression levels after MPP^+^ exposure in both WT and *Ask1*-deficient mice, which were consistent with the log10 ratios observed in the RNA sequencing analysis depicted in Figure 11D. Specifically, the log10 ratios and qRT-PCR analysis revealed that both WT and *ASK1*-deficient mice exhibited a decrease in *H2-q9* expression in response to MPP^+^. However, contrasting expression patterns were observed for *Depdc1b* and *Srp54b*, with an increase in expression observed in WT mice and a decrease in *Ask1*-deficient mice (Figure 11E).

To further investigate the impact of *Ask1* deficiency on MPP^+^-induced gene expression in WT and *Ask1*^−/−^ mice, we conducted a protein–protein network analysis of the detected DEGs within Cytoscape using STRING (Supplementary Figure S9A). With the aid of the ClusterONE plugin for Cytoscape, we isolated two clusters deemed most significant (cluster 1: *p* = 3.336 × 10^−4^; cluster 2: *p* = 6.789 × 10^−4^). Notably, the DEGs in cluster 1 were enriched in the inflammatory response pathway (BP, *p* = 2.91 × 10^−16^) (Supplementary Figure S9B), indicating a pronounced modulatory effect of *Ask1* on MPP^+^. On the other hand, the DEGs in cluster 2 were significantly associated with synaptic transmission (BP, *p* = 1.64 × 10^−6^) (Supplementary Figure 9C). Of particular interest, *Slc5a7* was among the genes included in cluster 1, suggesting a potential underlying mechanism for the neuroprotective effects of *Ask1* deficiency against MPP^+^ toxicity (Supplementary Figure 9C).

**Figure 12:**
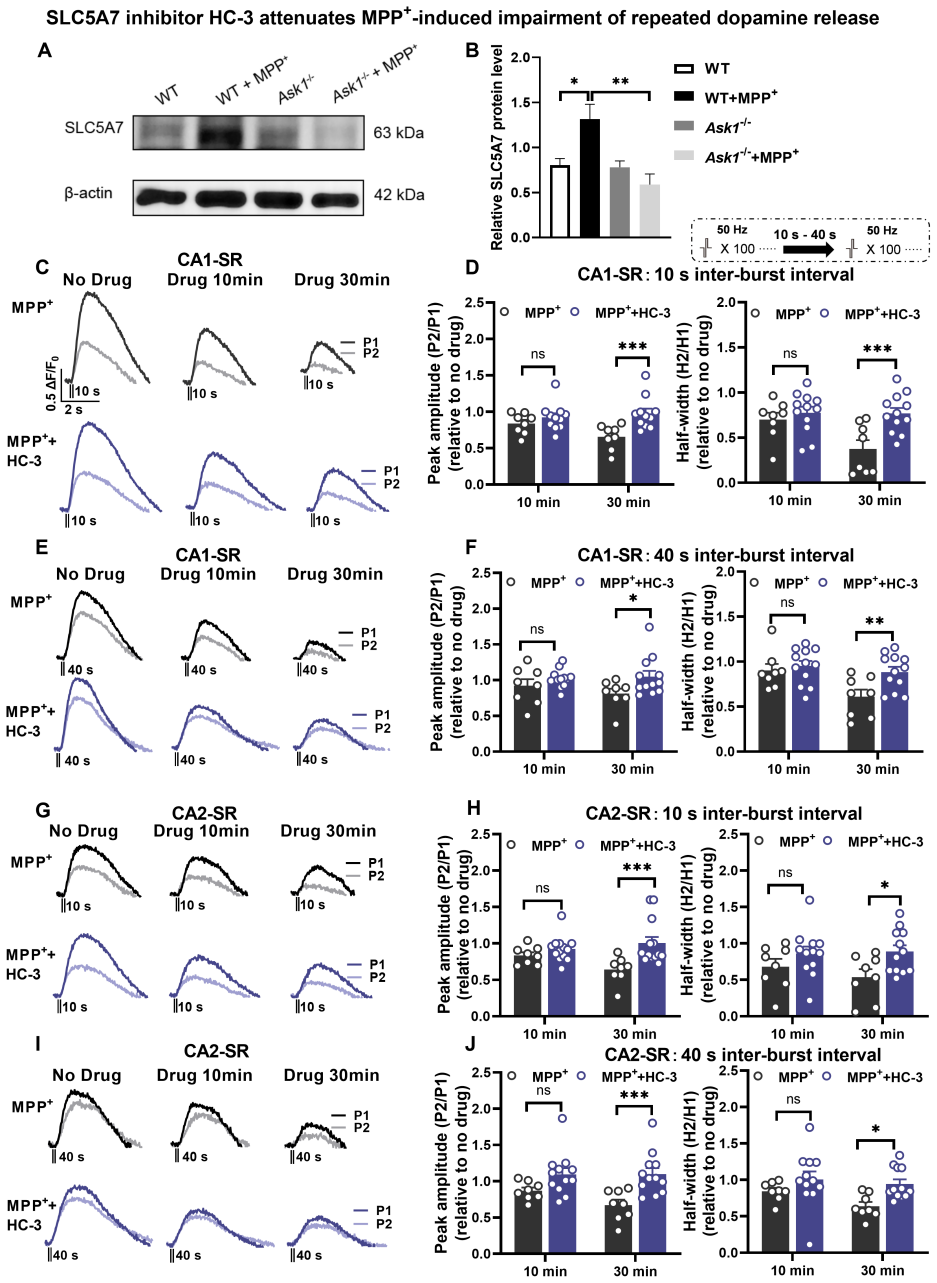
SLC5A7 inhibitor HC-3 rescues MPP^+^-induced impairment of repeated dopamine release in the CA1-SR and CA2-SR regions. **(A,B)** Protein expression changes in hippocampal SLC5A7 were examined in *Ask1*^−/−^ and littermate mice. Protein expression changes were compared to the littermate group treated with MPP^+^. One-way ANOVA was used for statistical analysis: ^*^p < 0.05, ^**^p < 0.01; *n* = 5 mice. The stimulation protocol is inserted. **(C,E)** Representative fluorescence traces in the CA1-SR region in response to paired-burst stimulation with **(C)** 10-s and **(E)** 40-s inter-burst intervals for MPP^+^ alone (black lines) and MPP^+^ + HC-3 experiments (red lines). **(D,F)** The bar graphs present the paired-fluorescence peak ratio (P2/P1; left) and the half-width ratio (right) of MPP^+^ (black bar) and MPP^+^ + HC-3 (red bar) for **(D)** 10-s and **(F)** 40-s inter-burst intervals. **(G,I)** Representative fluorescence traces from MPP^+^ alone and MPP^+^ + HC-3 experiments in response to **(G)** 10-s and **(I)** 40-s inter-burst stimulation in the CA2-SR region. **(H,J)** Bar graphs summarize the peak and half-width ratio values of fluorescence signals elicited by **(H)** 10-s and **(J)** 40-s inter-burst interval stimulations for the experimental groups in the CA2-SR region. Significant differences between experimental conditions are indicated by brackets and asterisks using *t*-tests: ^*^p < 0.05, ^**^p < 0.01, ^***^p < 0.001. The number of data points in the graphs corresponds to the number of slices from four mice.

As a result, we conducted Western blot (WB) analysis (Figure 12A) to examine the protein expression levels of SLC5A7. The aim was to determine whether MPP^+^ is capable of inducing SLC5A7 expression at the protein level and whether this induction is hindered in *Ask1*^−/−^ mice. The experimental results revealed a significant increase in SLC5A7 expression in response to MPP^+^ in WT mice, while no such increase was observed in *Ask1*^−/−^ mice following the application of MPP^+^ (Figures 12A,B).

**Figure 13:**
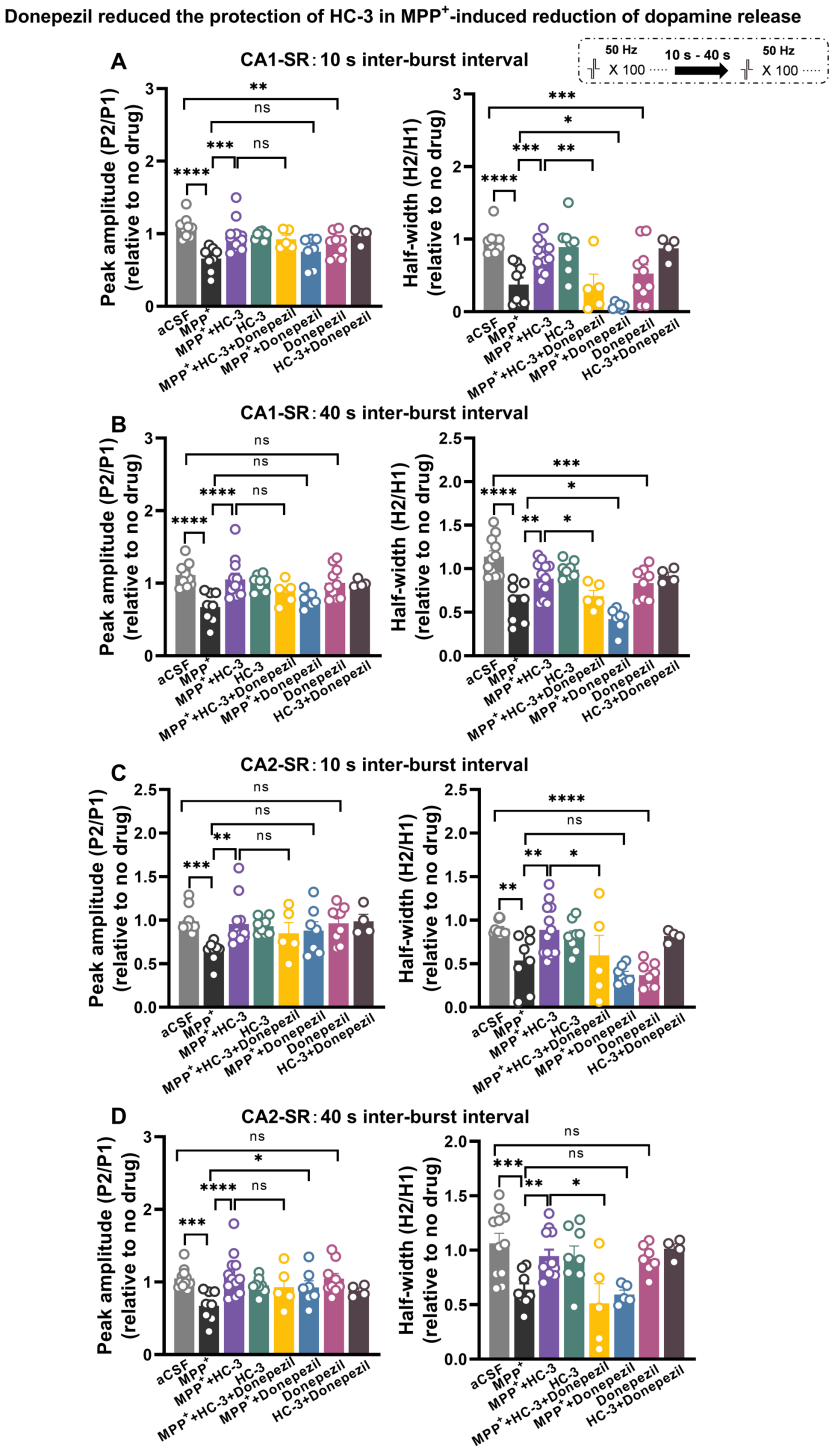
Acetylcholinesterase (AchE) inhibitor donepezil reduced the protection of HC-3 in MPP^+^-induced reduction of dopamine release in the CA1-SR and CA2-SR regions. Bar graphs illustrate the effects of different drugs and drug combinations with 10-s interval **(A)** and 40-s interval **(B)** on peak value ratio fluorescence (left) and half-width (right) in the CA1-SR region and **(C,D)** in the CA2-SR region. The stimulation protocol is inserted. Statistical significance is indicated by brackets and asterisks (Fisher’s LSD test): ^*^p < 0.05, ^**^p < 0.01, ^***^p < 0.001, ^****^p < 0.0001. The number of data points in the graphs corresponds to the number of slices from four mice.

Based on RNA sequencing, qRT-PCR, and WB data about the MPP^+^ effects on *Slc5a7* and SLC5A7 expression, we explored the functional consequences of altered SLC5A7 functionality. To this end, we used hemicholinium-3 (HC-3) (Lau et al., 2013), a known antagonist of SLC5A7 (CHT1), to determine whether it could rescue the impairment of dopamine release incubation with MPP^+^. Our findings demonstrated that 15 μM HC-3 significantly improved dopamine release evoked by paired-burst stimulation 30 min after MPP^+^ incubation in both CA1-SR and CA2-SR (Figures 12C–J). The application of HC-3 via bath did not result in any significant alteration in dopamine release in the CA1-SR and CA2-SR regions compared to the measurements without any drug, as depicted in Figures 13A–D and Supplementary Figure S10A. Thus, we further confirmed that SLC5A7 may play a crucial role in the protective effect of *Ask1*^−/−^ against MPP^+^.

We speculated that *Ask1* deficiency may affect the balance or interaction between dopamine and Ach under MPP^+^ incubated conditions. To investigate the impact of increased acetylcholine levels on dopamine release, we used the acetylcholinesterase (AchE) inhibitor donepezil and tested its effect on the paired-burst stimulation-induced dopamine release inhibition at 10- and 40-s IBIs (see schematic representation in Figure 13). Figure 13 illustrates the effects observed in the CA1-SR region (Figures 13A,B). Following a 30-min bath application of 10 μM Donepezil, a significant decrease in the normalized peak and half-width ratios at 10-s IBI was observed compared to the aCSF group. Notably, when MPP^+^ and Donepezil were applied together, a significant enhancement in reducing the 10-s and 40-s paired-pulse fluorescence half-width ratio was observed, surpassing the effects seen with MPP^+^ incubation alone. Furthermore, it’s important to note that the co-administration of Donepezil, HC-3, and MPP^+^ diminished the protective impact of HC-3 on the MPP^+^ paired-pulse fluorescence half-width ratio. Shifting focus to the CA2-SR region (Figures 13C,D), exposure to 10 μM Donepezil for 30 min solely resulted in a reduction of the 10-s paired-pulse fluorescence half-width ratio in comparison to the aCSF group. With the concurrent administration of MPP^+^ and Donepezil, a marked enhancement emerged in the peak ratio of the 10- and 40-s paired-pulse fluorescence, compared with MPP^+^ incubation alone. Contrary to the CA1-SR region, the presence of HC-3 during incubation did not exert any influence on this effect. In addition to the data presented in Figure 13, corresponding representative normalized fluorescence traces can be found in Supplementary Figure S10.

## 4 Discussion

This study elucidated the dynamics of evoked hippocampal dopamine release using a genetically encoded dopamine sensor alongside electrophysiological techniques under control conditions and MPP^+^ exposure in *Ask1*-expressing and *Ask1*-deficient mice. Comparative analysis of distinct hippocampal subregions revealed partial region-specific characteristics of dopamine release dependent on stimulation frequency or pulse number. Moreover, even in control slices, subsequent dopamine release events displayed severely attenuated signaling and slow recovery, indicating short-term depression. Furthermore, exposure to the PD-linked toxin MPP^+^ substantially impaired the recovery of dopamine release in response to paired-burst stimulation. Strikingly, the genetic deletion of *Ask1* facilitated the recovery of dopamine signaling under both control and MPP^+^-impaired conditions. Transcriptomic analysis using RNA sequencing identified differential gene expression patterns elicited by MPP^+^ in *Ask1*-deficient versus *Ask1*-intact mice. Notably, the removal of *Ask1* prevented the transcriptional increase in response of the choline transporter gene *Slc5a7* to MPP^+^ exposure. Experiments employing specific pharmacological inhibitors of *Slc5a7* and acetylcholinesterase indicated that *Slc5a7* participates in *Ask1* deficiency-mediated protection against MPP^+^ neurotoxicity. Thus, our data imply that modulation of the dopamine-acetylcholine equilibrium may constitute a mechanism underlying *Ask1* deficiency’s neuroprotective effects. Overall, this study illuminates the molecular underpinnings and functional consequences of *Ask1* deficiency on hippocampal dopamine release dynamics, particularly under neurotoxin-induced duress. These observations unveil previously undefined roles of *Ask1* in modulating neurotransmitter systems indispensable for cognitive function.

### 4.1 The spatiotemporal dynamics of dopamine release in hippocampal subregions

While previous research has explored dopamine release dynamics in regions such as the striatum (Cragg, 2003; Whittaker et al., 2008; Chen et al., 2019) and nucleus accumbens (Badrinarayan et al., 2012; Yorgason et al., 2014; Hill et al., 2018; Chen et al., 2019), limited investigations have examined hippocampal dopamine signaling across various synaptic plasticity contexts. The hippocampus receives dopaminergic inputs, and dopamine has been implicated in modulating hippocampal synaptic plasticity and cognitive functions. However, the precise spatiotemporal profile of dopamine release during different plasticity phenomena in hippocampal subregions remains ambiguous. To address this gap, we utilized the genetically encoded dopamine sensor dLight1 (Patriarchi et al., 2018) to monitor evoked dopamine release dynamics in acute hippocampal slices. Compared to approaches like microdialysis, dLight1.2 provides higher spatiotemporal resolution and enables the detection of rapid, cell-specific dopamine release (Muller et al., 2014; Jaquins-Gerstl and Michael, 2015; Ganesana et al., 2017; Patriarchi et al., 2018).

Our observations revealed that dopamine release shows a stronger dependency on pulse number compared to stimulation frequency or theta-burst patterns. Furthermore, different pulse stimulations resulted in greater dopamine discharge in CA1-SLM compared to CA1-SR and CA2-SR, indicating regional heterogeneity in release probability. Importantly, a second dopamine release event that closely followed the initial discharge exhibited significantly attenuated signaling, suggesting robust paired-burst depression. This depression demonstrated varying recovery kinetics across hippocampal subregions, lasting over 60 s in CA1-SR/CA2-SR but recovering within 60 s in CA1-SLM. The occurrence of depression and slow-release recovery suggests reduced dopamine signaling during repeated hippocampal activation, which may have implications for modulating synaptic plasticity during learning tasks that require sustained activity.

The hippocampus receives dopaminergic input from several regions, including the VTA, SNc, and LC. The VTA (Lisman and Grace, 2005) and SNc (Tsetsenis et al., 2021) are the main sources of dopaminergic modulation in the hippocampus, projecting predominantly to CA1, subiculum, and dentate gyrus. On the other hand, the LC (Kempadoo et al., 2016; Takeuchi et al., 2016) provides more diffuse dopaminergic innervation throughout the hippocampus. The heterogeneous distribution of dopaminergic fibers from distinct origins, in addition to the variability in release dynamics and postsynaptic responses, contributes to diverse dopamine signaling mechanisms across hippocampal circuits. This allows dopamine to modulate specific aspects of hippocampal function in a subregion-specific manner. Furthermore, differences in release probability, vesicle pool size, and recycling kinetics in dopaminergic fibers from different origins likely contribute to the regional variation in recovery rate following paired-burst depression. Our findings on paired-burst dopamine release depression align with observations in the striatum (Condon et al., 2019), where high-probability varicosities exhibit short-term depression attributed to vesicle depletion. These variations may include differences in dopaminergic innervation density across hippocampal subregions and distinct synaptic architecture and circuitry in CA1/CA2 versus CA1-SLM microdomains. Whether upstream regulators of dopamine synthesis and packaging are regulated in a region-specific manner remains an open question. In summary, this investigation elucidates previously undefined relationships between hippocampal dopamine release, stimulation patterns, and inter-burst intervals across synaptic plasticity contexts and subregions.

In addition, it is indeed important to note that the striatum and hippocampus have distinct neural anatomy and functions. The striatum is primarily associated with motor control and reward behavior, whereas the hippocampus is involved in learning and memory consolidation. Consequently, it is possible that there are differences in the regulation of dopamine release between these two brain regions. Exploring these differences could provide valuable insights into the unique functional roles of dopamine in the striatum and hippocampus. Thus, it is important for future research to further elucidate the similarities and differences in the regulation of dopamine release between the striatum and hippocampus.

### 4.2 *Ask1* deletion attenuates acute dopamine dysfunction induced by MPP^+^

Our objective was to identify novel molecular targets capable of modulating the alterations in dopamine release provoked by MPP^+^. To achieve this, we investigate the response of hippocampal dopamine signaling to varied stimulation protocols in *Ask1*-deficient mice. Our findings demonstrate that the *Ask1* deletion amplifies dopamine release elicited by diverse stimulation protocols, even under control conditions. Interestingly, we observed that *Ask1* deficiency not only enhances dopamine discharge induced by protocols known to potentiate synaptic transmission but also differentially elevates the dependence on pulse and low-frequency dopamine release across all examined hippocampal subregions. The facilitation of dopamine release by *Ask1* deletion varies regionally, with a more significant augmentation observed in CA2-SR compared to other areas, potentially suggesting a more substantial contribution of *Ask1* to dopamine release recovery in the hippocampal CA2. However, further examination is warranted to better understand this relationship.

We discovered that *Ask1* deletion could rescue the impairment of extracellular and vesicular dopamine release provoked by MPP^+^. However, the impact of *Ask1* deficiency differed between these two aspects, with minimal effects on vesicular dopamine release observed over a 40-s period, particularly in CA2. This suggests that *Ask1* deletion may not directly regulate dopamine vesicle release itself but instead modify aspects of dopamine recycling and reloading. This modulation could potentially occur through its influence on the expression of the choline transporter and/or other unidentified mechanisms rather than directly affecting dopamine transporter expression, as indicated by the RNA sequencing results.

To identify potential *Ask1*-dependent gene expression changes under MPP^+^ exposure, we performed RNA sequencing on hippocampal slices from wild-type and *Ask1*-null mice, both with and without MPP^+^ treatment. Our RNA sequencing analysis revealed that the gene *Slc5a7* plays a critical role in mediating *Ask1* deletion’s protective effects against MPP^+^. We observed that *Slc5a7* participates in the pathways affected by MPP^+^ in both wild-type and *Ask1*-deficient mice. Remarkably, the use of the SLC5A7 inhibitor HC-3 demonstrated protective effects on MPP^+^-impaired dopamine release similar to *Ask1* deletion. Considering that *Slc5a7* mediates choline uptake for acetylcholine synthesis, inhibition of *Slc5a7* leads to lower acetylcholine levels. Our results suggest an inverse correlation between dopamine release and acetylcholine levels. Therefore, the protective mechanism of *Ask1* deletion against MPP^+^ toxicity may involve reducing acetylcholine levels in the hippocampus, thereby restoring dopamine release.

Our finding that *Slc5a7* inhibition rescues MPP^+^-induced dopamine release deficits provides evidence for the exploitative nature of antagonistic dopamine/acetylcholine interactions in the context of *Ask1* deletion. Nicotinic acetylcholine receptors (nAChRs) have been reported to be highly expressed in midbrain dopaminergic cells and play a crucial role in regulating dopamine neural activity (Faure et al., 2014). AChRs on striatal DA axons are known to enhance dopamine release evoked by single-pulse or low-frequency stimulation while suppressing release evoked by high-frequency stimulation (Rice and Cragg, 2004; Zhang and Sulzer, 2004). Hence, an elevated acetylcholine level could potentially suppress hippocampal dopamine release evoked by 50 Hz stimulation via nAChRs on dopamine terminals. Future investigations monitoring ACh levels would be warranted to further explore this mechanism. *Ask1* deletion may attenuate this suppression and compensate for MPP^+^-mediated dopamine deficits, thus suggesting that restoring dopamine/acetylcholine balance could be a potential therapeutic approach for dopaminergic toxicity and Parkinson’s disease. However, achieving optimal modulation of each neurotransmitter requires further study. Notably, while enhancing acetylcholine is a current strategy for Alzheimer’s disease, our results suggest that such enhancement might negatively impact hippocampal dopamine release, which is also crucial for memory and learning.

### 4.3 The dopamine-acetylcholine dependency in MPP^+^ toxicity and *Ask1*^−/−^-mediated protection

Our data demonstrated that the SLC5A7 antagonist HC-3 significantly improved paired-pulse dopamine release recovery 30 min after MPP^+^ exposure in both CA1-SR and CA2-SR, thus validating the pivotal role of SLC5A7 in mediating the protective effects of *Ask1* deletion against MPP^+^. Based on our findings, we hypothesized that *Ask1* deficiency may impact the equilibrium and interactions between dopamine and acetylcholine. To assess this hypothesis, we employed donepezil, an acetylcholinesterase inhibitor, to examine the effects of elevated acetylcholine levels on dopamine release. The results revealed that co-administration of MPP^+^ and donepezil substantially reduced the paired-burst peak ratio and paired-burst half-width compared to MPP^+^ alone. However, this effect was mitigated by HC-3 administration. Additionally, the protective effects of HC-3 on MPP^+^-induced half-width decreases were attenuated when donepezil was applied in combination. These observations suggest that the *Ask1* deletion plays a role in regulating the balance between dopamine and acetylcholine under MPP^+^ neurotoxicity.

In line with our findings, a previous study demonstrated that the compound DA-9805 restores motor function by normalizing elevated striatal acetylcholine levels through modulating dopamine transmission and choline acetyltransferase expression while also providing neuroprotection (Huh et al., 2022). Other investigations have reported increased acetylcholine levels in Parkinson’s disease (PD) models. The loss of striatal dopamine in PD can stimulate cholinergic interneurons, leading to elevated acetylcholine levels (Ztaou and Amalric, 2019). However, postmortem analyses of PD patient hippocampi have shown a significant loss of the cholinergic receptor CHRM1 (Sabbir et al., 2022). *In vivo* neuroimaging using PET has revealed reduced striatal dopamine signaling, which enhances the excitability and synaptic reorganization of cholinergic interneurons, resulting in increased acetylcholine release (Sanchez-Catasus et al., 2022). Conversely, other studies have indicated that dopamine deficiency can lead to a reduction in acetylcholine levels (Zurkovsky et al., 2013; Tanimura et al., 2018; McKinley et al., 2019).

Our investigation provides valuable insights into the potential interactions between dopamine and acetylcholine within the hippocampus, highlighting the significant regulatory role of SLC5A7. The experimental evidence obtained through the use of HC-3 and donepezil to elucidate the influence of SLC5A7 and acetylcholine on dopamine release represents a notable strength of our study. While our observations offer promising clues and implications, further investigation is imperative in animal models and human subjects to determine the translational relevance and potential therapeutic applications of these findings.

## 5 Conclusion

This study provides crucial insights into the intricate mechanisms by which *Ask1* deletion protects against dopamine signaling impairments caused by the PD-linked neurotoxin MPP^+^ in the hippocampus. We elucidate the complex interplay between hippocampal dopamine release dynamics, MPP^+^ excitotoxicity, and *Ask1* expression. Our data highlight *Ask1* deficiency as a novel intervention capable of mitigating early hippocampal damage induced by MPP^+^, potentially through modulating the transcriptional response of the acetylcholine regulator SLC5A7 to MPP^+^ exposure. Moreover, we shed light on the link between dopamine and acetylcholine, underscoring the significance of maintaining a balance between these neurotransmitters. These findings profoundly enhance our understanding of *Ask1*’s role in modulating hippocampal dopamine release and gene expression in pathological states relevant to neurodegeneration.

## Data availability statement

The original contributions presented in the study are included in the article/Supplementary material, further inquiries can be directed to the corresponding author/s. The data presented in the study are deposited in the BioProject repository, accession number PRJNA1045080.

## Ethics statement

The animal study was approved by the Institutional Animal Care and Use Committee of Fudan University, Shanghai Medical College. The study was conducted in accordance with the local legislation and institutional requirements.

## Author contributions

FZ: Conceptualization, Data curation, Formal analysis, Investigation, Methodology, Project administration, Software, Validation, Writing – original draft, Writing – review & editing. CHL: Project administration, Writing – review & editing. YHZ: Writing – review & editing. YY: Project administration, Writing – review & editing. YQG: Writing – review & editing. TB: Conceptualization, Funding acquisition, Supervision, Writing – review & editing.
