# Supplementary material for: Apoptosis signal-regulating kinase 1 (Ask1) deficiency alleviates MPP+-induced impairment of evoked dopamine release in the mouse hippocampus
Source: Front Cell Neurosci. 2024 Feb 13;18:1288991. doi: 10.3389/fncel.2024.1288991 (PMC10896914; doi:10.3389/fncel.2024.1288991)
Supplement: Supplementary file 1 [file Presentation_1.pdf]

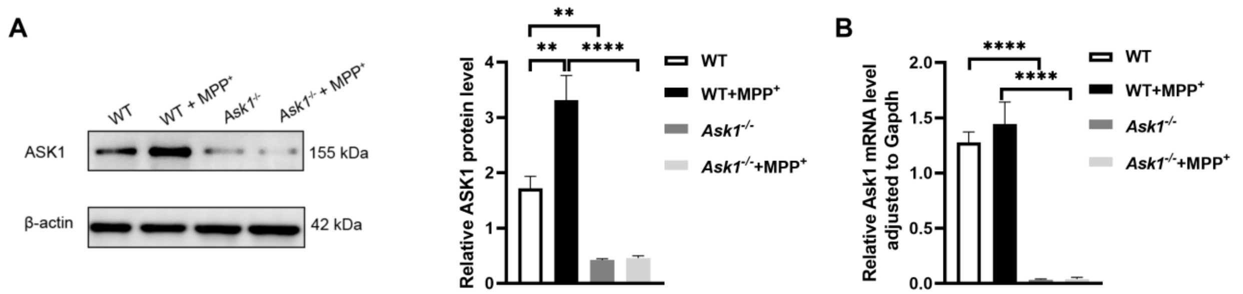

Supplementary Figure 1. Expression of ASK1 protein and *Ask1* mRNA following tamoxifen administration and MPP$^+$ exposure. (A) Hippocampal ASK1 protein expression was assessed in transgenic mice one week after tamoxifen administration (*Ask1*$^{-/-}$) and saline administration (littermates: WT). The effect of MPP$^+$ application on ASK1 expression in acute hippocampal slices of the two mouse groups is shown in the representative western blot (left) and quantified in the bar graph (right, 5 mice for each condition). **(B)** Similarly, changes in hippocampal *Ask1* mRNA expression were analyzed in *Ask1*$^{-/-}$ mice and their littermates (6 mice for each group). Tukey's multiple comparisons test revealed statistical significance indicated with asterisks and brackets: ** $P < 0.01$, **** $P < 0.0001$.

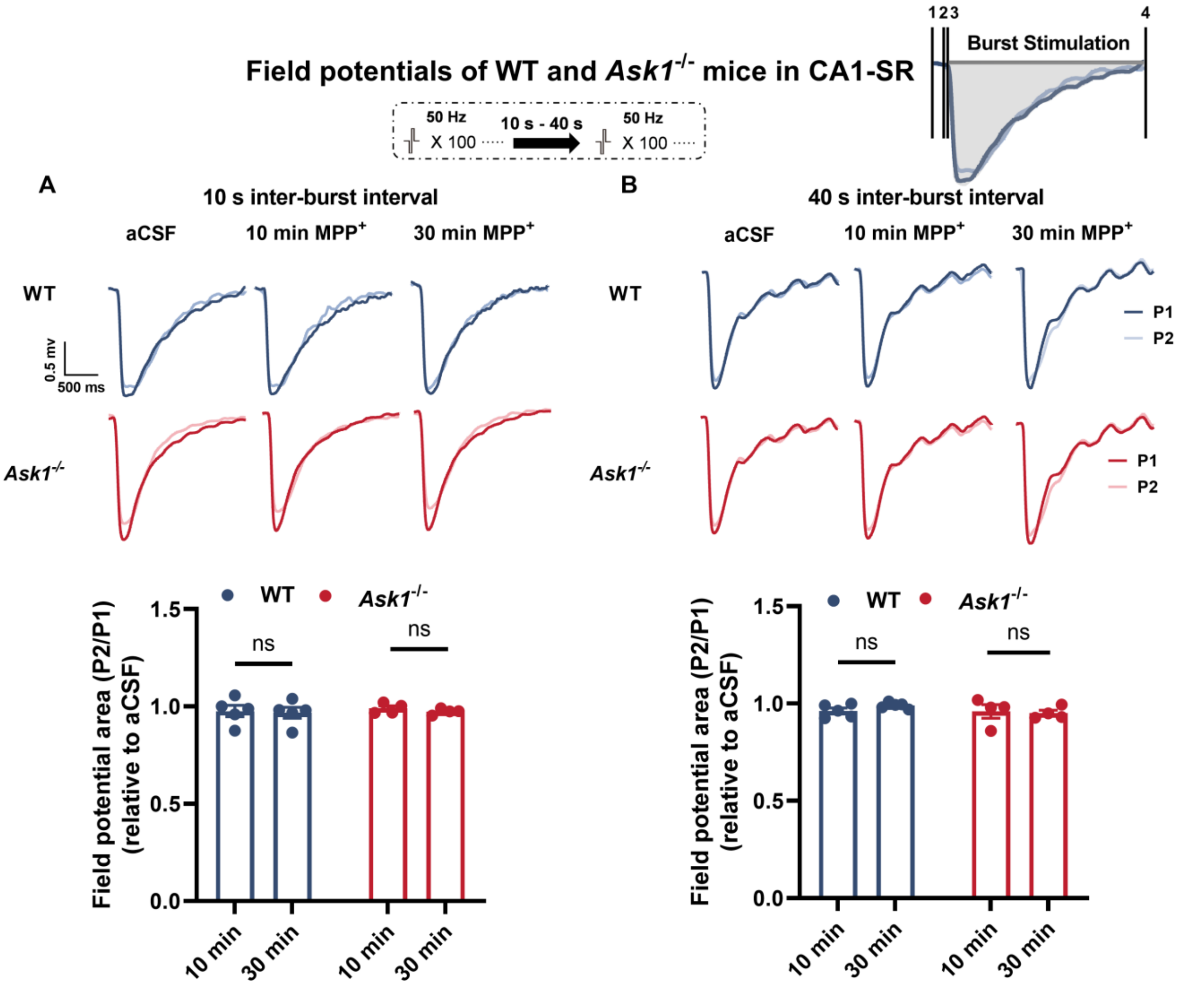

Supplementary Figure 2. MPP$^+$ bath application does not affect the area of paired-burst (50 Hz with 100 pulses) stimulation-induced field potentials in WT and *Ask1$^{-/-}$* hippocampal CA1-SR. (A-B) Graphs presenting the ratio of the areas of the two burst-induced field potentials for inter-burst intervals of 10 s (A) and 40 s (B) in WT and *Ask1$^{-/-}$* hippocampal CA1-SR, both before and during MPP$^+$ application. Number of data points in the graphs corresponds to the number of slices from 4 mice. Insets above depict representative field potentials in response to the paired bursts. The insert graph showed the measurement of the signal: 1,2,3,4 indicate the markers and marker 1, 2 for baseline 3, 4 for signal. Horizontal line and gray color area indicate the stimulation area which measured by using Clampfit and for markers the baseline was recognized to measure the area of the field potential between the maker 3 and 4.

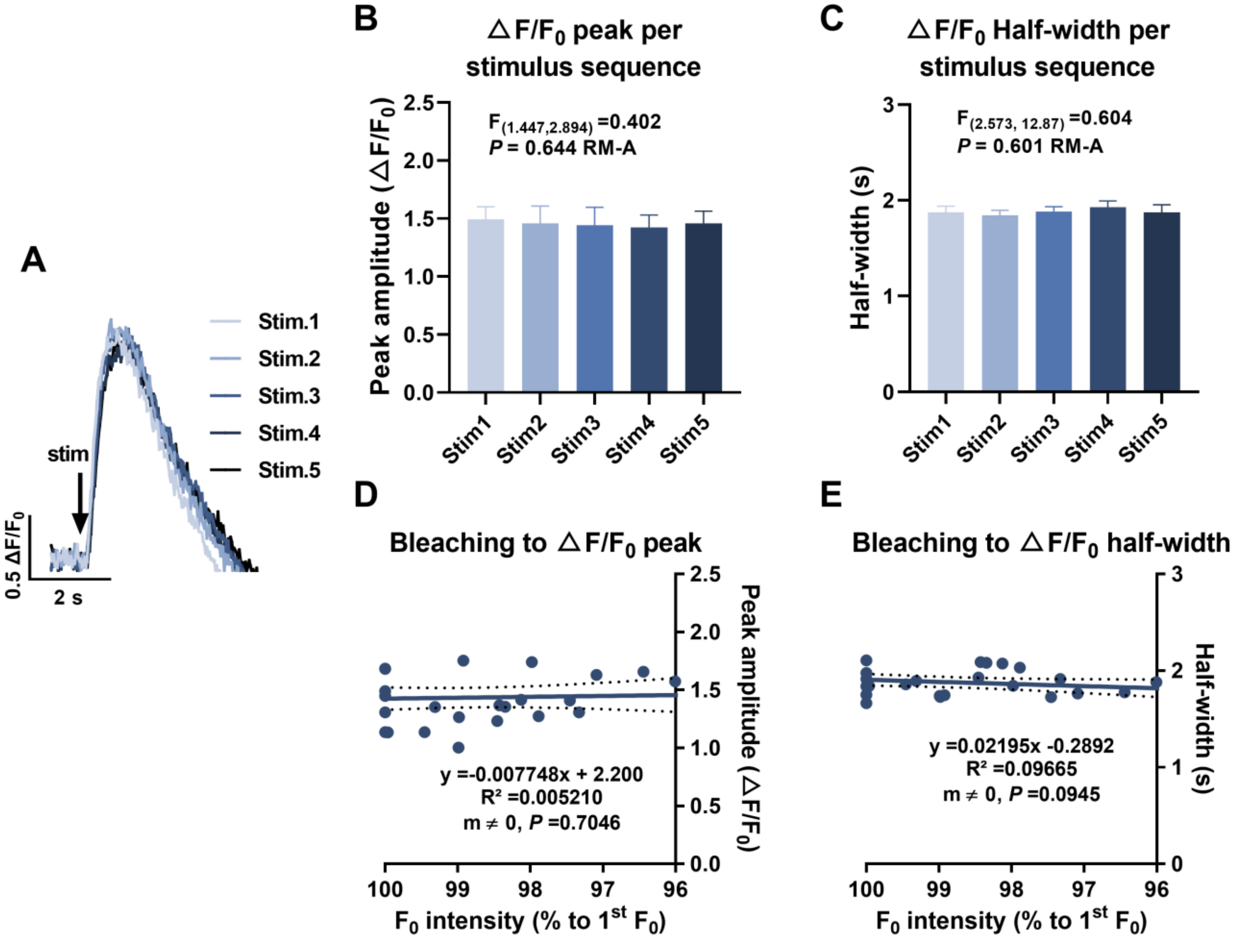

Supplementary Figure 3. Repeated electrical stimulations or bleaching do not affect the peak or half-width dynamics of fluorescence changes. **(A)** Representative $\Delta F/F_0$ traces for a sequence of stimulations at constant intervals and pulses per burst. **(B)** Bar graphs showing peak amplitudes at the stimulation sequences (Stim1 to Stim5), where each stimulation consisted of the same number of pulses per burst. The sequence of stimulations does not alter the resulting peak amplitude, as confirmed by RM-OW-ANOVA: $F_{(1.447,\ 2.894)} = 0.402$, $P = 0.644$. **(C)** Similar observations were made for the half-width values, as indicated by RM-OW-ANOVA: $F_{(2.573,\ 12.87)} = 0.6$, $P = 0.6$. **(D-E)** Scatter plots depicting the relationship between bleaching and peak amplitude (D) and half-width (E) to assess potential effects of basal reduction in fluorescence intensity due to bleaching. The degree of bleaching was assessed by normalizing F_0 values before every stimulation to the very first one. Linear regression analysis does not reveal a significant difference in the slope from m = 0.

Time-Lapse Fluorescence Imaging of 100 Hz Stimulation Responses with Varying Pulse Numbers

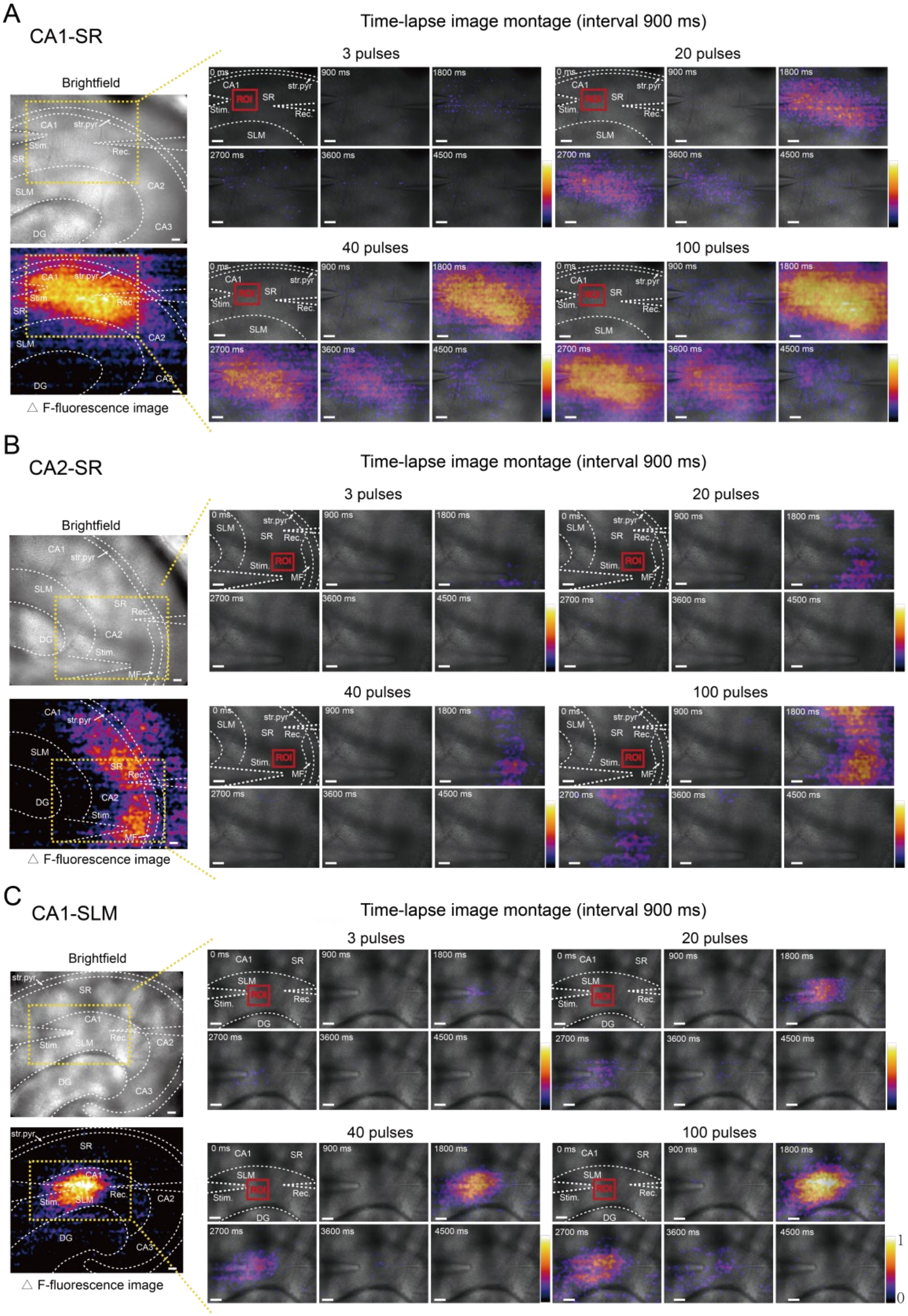

Supplementary Figure 4. Time series of fluorescence changes induced with different numbers of pulses per burst in three distinct regions of hippocampal slices, as presented in Figure 3. (A) The brightfield image indicates the location of layers and electrodes. Below, a ΔF-fluorescence image is displayed. The yellow dotted box highlights the zoomed-in area, which is further presented in the time series montage on the right. Consecutive time-lapse $\Delta F/F_0$ values are color-coded and overlaid onto the brightfield image. The montages depict time-lapse recordings at 3, 20, 40, and 100 pulses per burst in the CA1-SR region. A red box indicates the commonly used positions of the region of interest (ROI) from which fluorescence intensity values were acquired. **(B-C)** Similar representations of fluorescence changes are shown for the CA2-SR region in (B) and for the CA1-SR region in (C). Horizontal scale bars: 100 µm; str. pyr.: stratum pyramidale; SR: stratum radiatum; SLM: stratum lacunosum-moleculare; MF: mossy fiber; DG: dentate gyrus; Stim.: stimulation electrode; Rec.: recording electrode; ROI: region of interest.

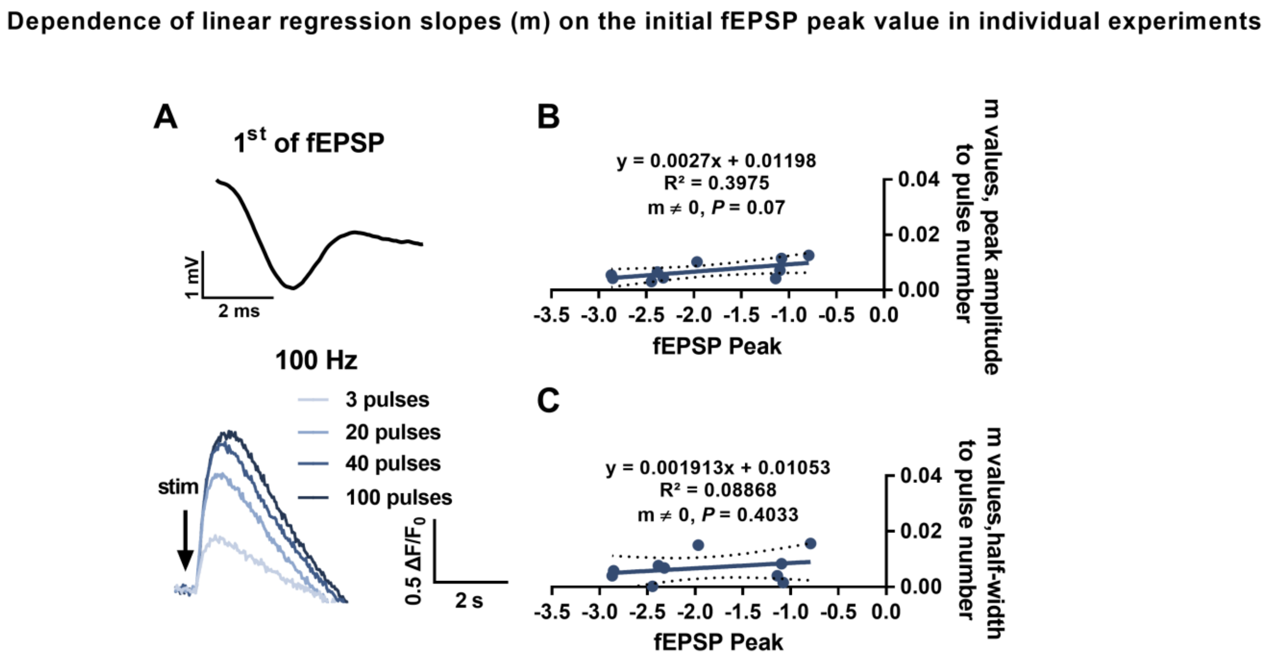

Supplementary Figure 5: Effect of initial fEPSP size on linear regression slopes in experiments with varied pulse numbers per burst. (A) An example of an initial fEPSP of a burst in the CA1-SR region, consisting of 3 pulses, is shown. The size of such fEPSPs is considered representative of the stimulation strength achieved in individual experiments involving a sequence of stimulations. The applied stimulation sequence ranged from 3 to 100 pulses at 100 Hz. The normalized fluorescence changes corresponding to the sequence of indicated stimulation types are displayed below. **(B)** The scatter plot illustrates the relationship between the individual linear regression slopes (denoted as m) obtained from the experiments depicted in Figure 3C and the corresponding initial fEPSP size. The linear regression analysis was conducted to determine if a significant difference existed between the new slope value and zero. The analysis established that no significant difference was found. **(C)** Similar presentation of data and analysis for the dependence of linear regression slopes (m) obtained in Figure 3 for half-width values on initial fEPSP size is presented.

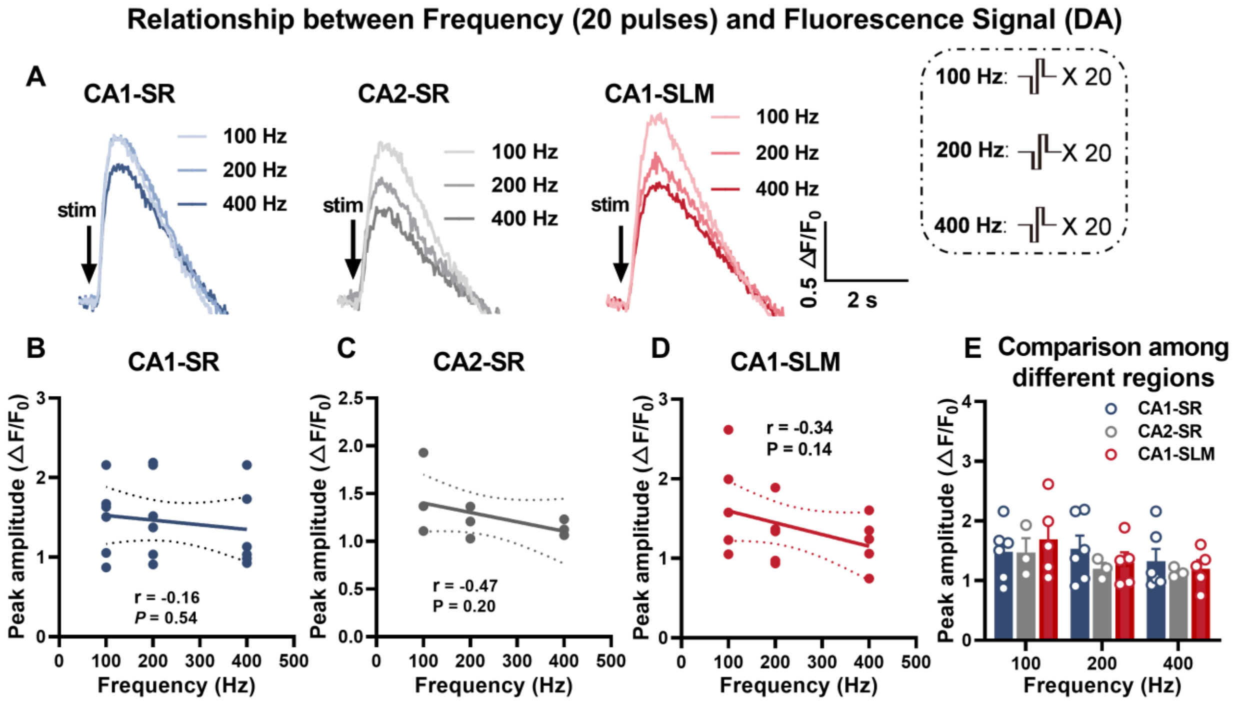

Supplementary Figure 6. Dependency of dopamine release on stimulation frequency in different hippocampal regions. (A) Averaged fluorescence traces in response to different frequencies of 40 subsequent stimuli within the CA1-SR, CA2-SR, and CA1-SLM regions. A stimulation protocol schema has been inserted. **(B-D)** Scatter plots displaying individual data points of $\Delta F/F_0$, along with the linear regression line and confidence interval. Pearson's correlation coefficient (r) and the corresponding p-values are indicated in each correlation plot. **(E)** Visual comparison of the averaged peak amplitude for different stimulation frequencies among hippocampal areas. Statistical analysis using RM-TW-ANOVA revealed no significant effect of regions on peak amplitude ($P = 0.63$, $F_{(2,33)} = 0.46$). Tukey's multiple comparisons test did not show any significant differences among the regions. Number of data points in the graphs corresponds to the number of slices from 4 mice. Stim: time point of stimulation; SR: stratum radiatum; SLM: stratum lacunosum-moleculare.

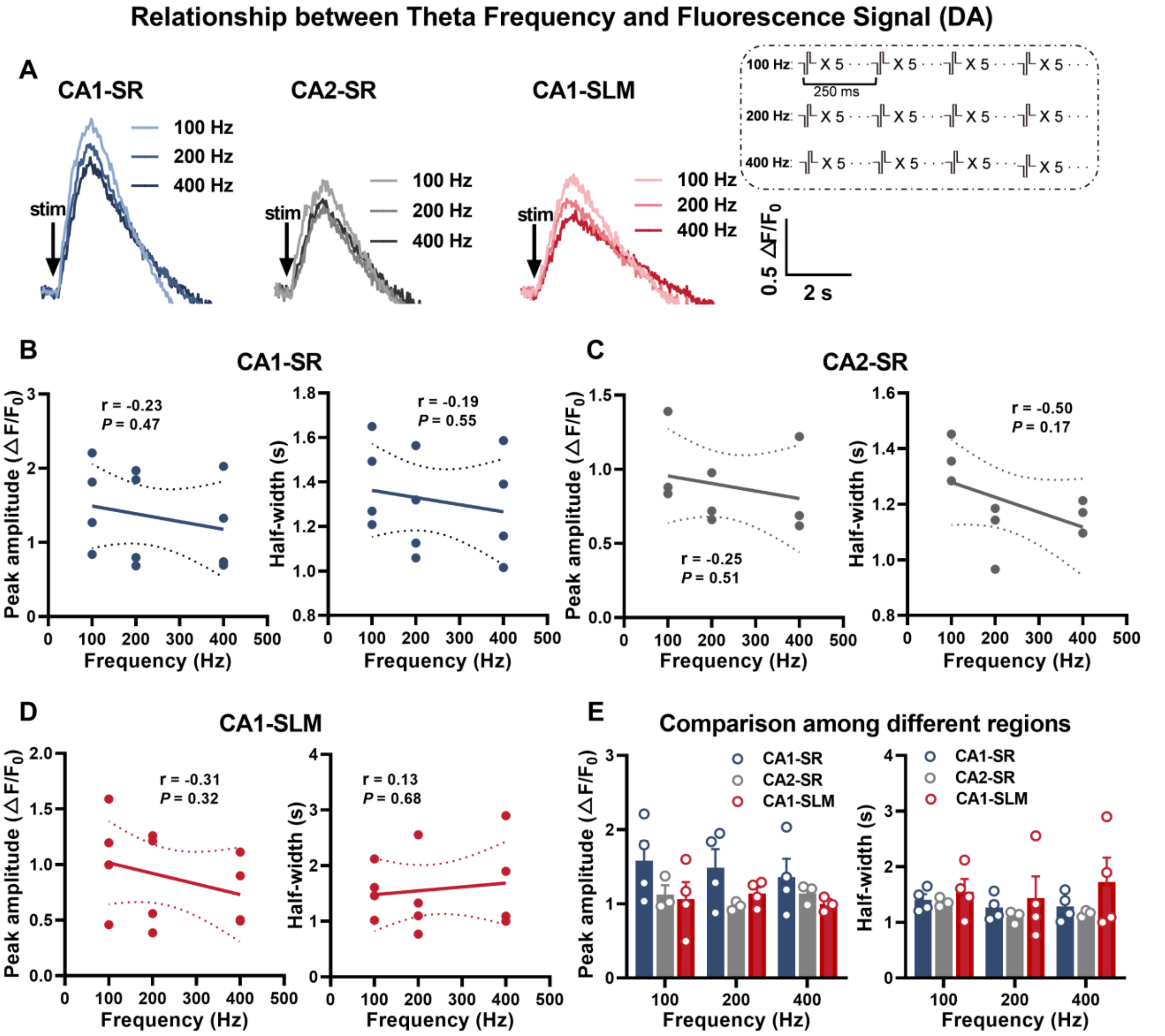

Supplementary Figure 7. Effect of burst frequency variations in theta burst stimulation on dopamine release (A) Averaged fluorescence traces showing the response to four bursts with five stimuli at frequencies ranging from 100 to 400 Hz, with an inter-burst interval of 250 ms, in the hippocampal CA1-SR, CA2-SR, and CA1-SLM regions. A stimulation protocol schema has been inserted. **(B-D)** Scatter plots displaying the peak $\triangle F/F_0$ value (left) and half-width value (right), along with linear regression and confidence intervals. The Pearson's correlation coefficient (r) and corresponding p-values are indicated in each plot. **(E)** Comparison of dopamine release among different regions based on the theta burst protocol. RM-TW-ANOVA analysis revealed a significant effect of regions on peak amplitude (P = 0.02, $F_{(2,24)}$ = 4.675 (peak amplitude); and P = 0.21, $F_{(2,24)}$ = 1.696 (Half-width). Tukey's post-hoc test multiple comparisons showed no significant difference among regions. The number of data points in the graphs corresponds to the number of slices from 4 mice. Abbreviations as in Supplementary Figure 6.

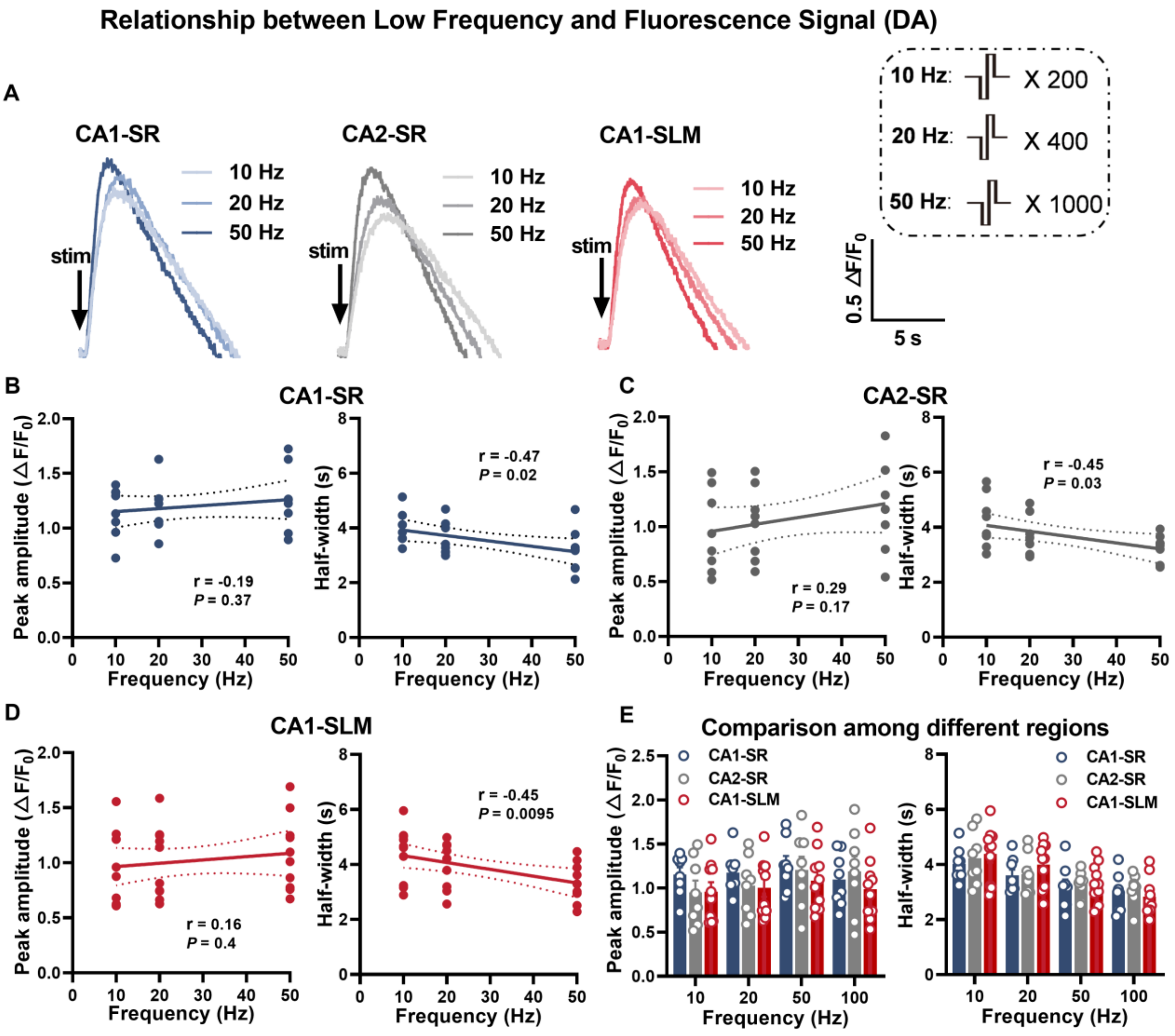

Supplementary Figure 8. Dependency of dopamine release on low-frequency stimulation in different hippocampal regions. (A) Averaged fluorescence traces in response to 100 subsequent stimuli at frequencies ranging from 10 to 50 Hz within CA1-SR, CA2-SR, and CA1-SLM regions are shown. A stimulation protocol schema has been inserted. **(B-D)** Scatter plots depicting individual data points, along with the linear regression and confidence interval, of peak $\Delta F/F_0$ (left) and half-width (right) values in CA1-SR (B), CA2-SR (C), and CA1-SLM (D) regions. The Pearson's correlation coefficient (r) and corresponding p-values are indicated in each correlation plot. **(E)** Visual comparison of the averaged peak and half-width values among different hippocampal regions. RM-TW-ANOVA analysis indicated no significant effect of regions on peak amplitude (P = 0.1292, $F_{(2,92)}$ = 2.093) and half-width (P = 0.5186, $F(2,92)$ = 0.6613). Tukey's post hoc test multiple comparisons showed no significant difference among the regions. The number of data points in the graphs corresponds to the number of slices from 4 mice. Abbreviations as in Supplementary Figure 6.

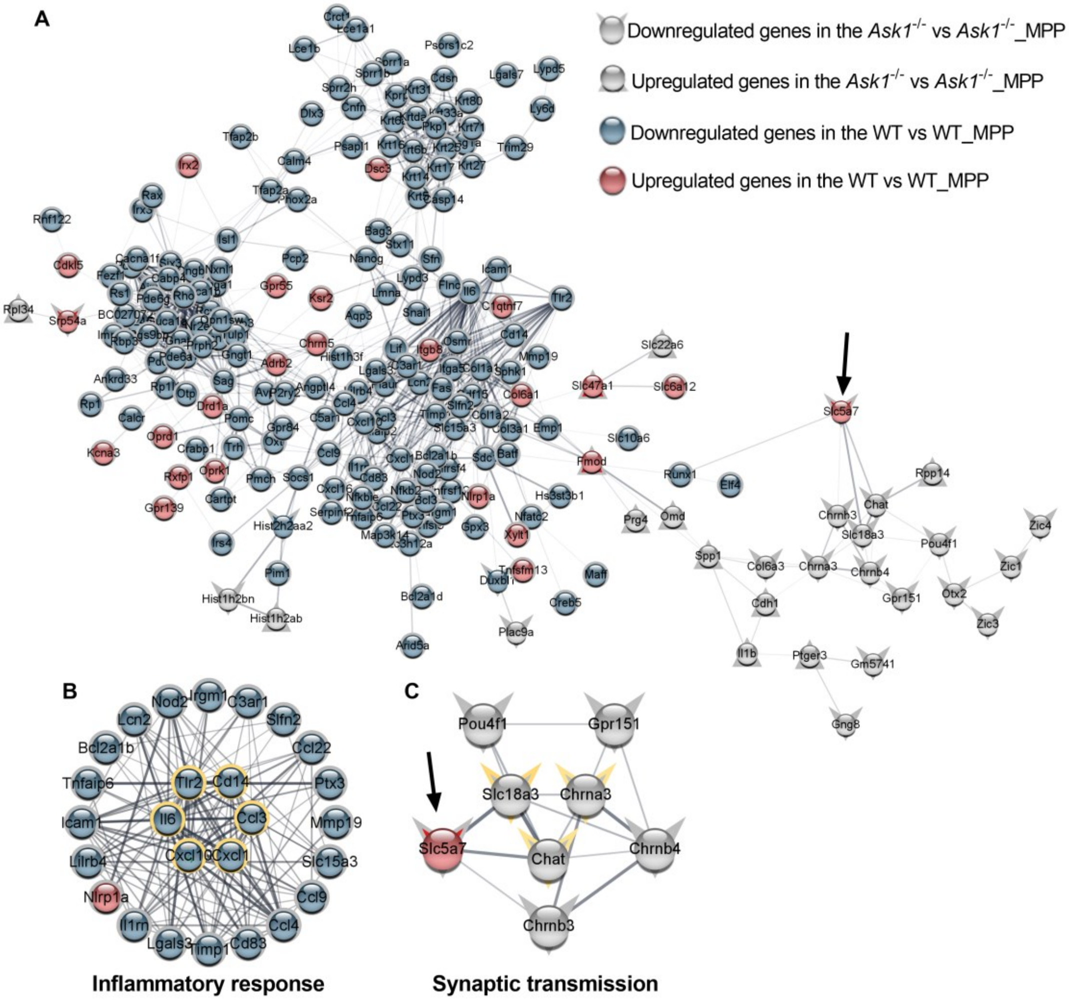

Supplementary Figure 9. Cluster analysis of the protein-protein interaction (PPI) network using Cytoscape based on detected DEGs. (A) The Venn diagram displays the overlap of 17 out of 359 DEGs identified in the comparison between WT vs. WT-MPP$^+$ (blue) and *Ask1*$^{-/-}$ vs. *Ask1*$^{-/-}$+MPP$^+$ (red). MPP$^+$ application resulted in a more pronounced alteration of gene expression in WT mice compared to *Ask1*-/- mice. The PPI network of the identified DEGs is represented by blue (downregulated) and red (upregulated) filled circles for the WT vs. WT-MPP$^+$ comparison. Gray filled circles present the DEGs for *Ask1*$^{-/-}$ vs. *Ask1*$^{-/-}$+MPP$^+$ (score > 0.4) comparison, where inverted triangles indicate downregulated DEGs and regular triangles indicate upregulated DEGs. Unconnected nodes were omitted. *Slc5a7* is indicated by a black arrow. **(B)** A PPI network illustrating the effect of *Ask1* deficiency on immune responses to MPP$^+$ application is shown. Cluster analysis isolated protein-protein interaction networks related to inflammatory responses. **(C)** A PPI network illustrating the effects of MPP$^+$ on DEGs related to synaptic transmission in the *Ask1*$^{-/-}$ vs. *Ask1*$^{-/-}$ + MPP$^+$ comparison is presented. *Slc5a7* is indicated by a black arrow. Thus, *Ask1* deficiency has a protective effect against immune responses to MPP$^+$. The networks were isolated using the ClusterONE plugin of Cytoscape and met the following criteria: node > 5, density > 0.5, quality > 0.5, and $P < 0.05$ (3 mice).

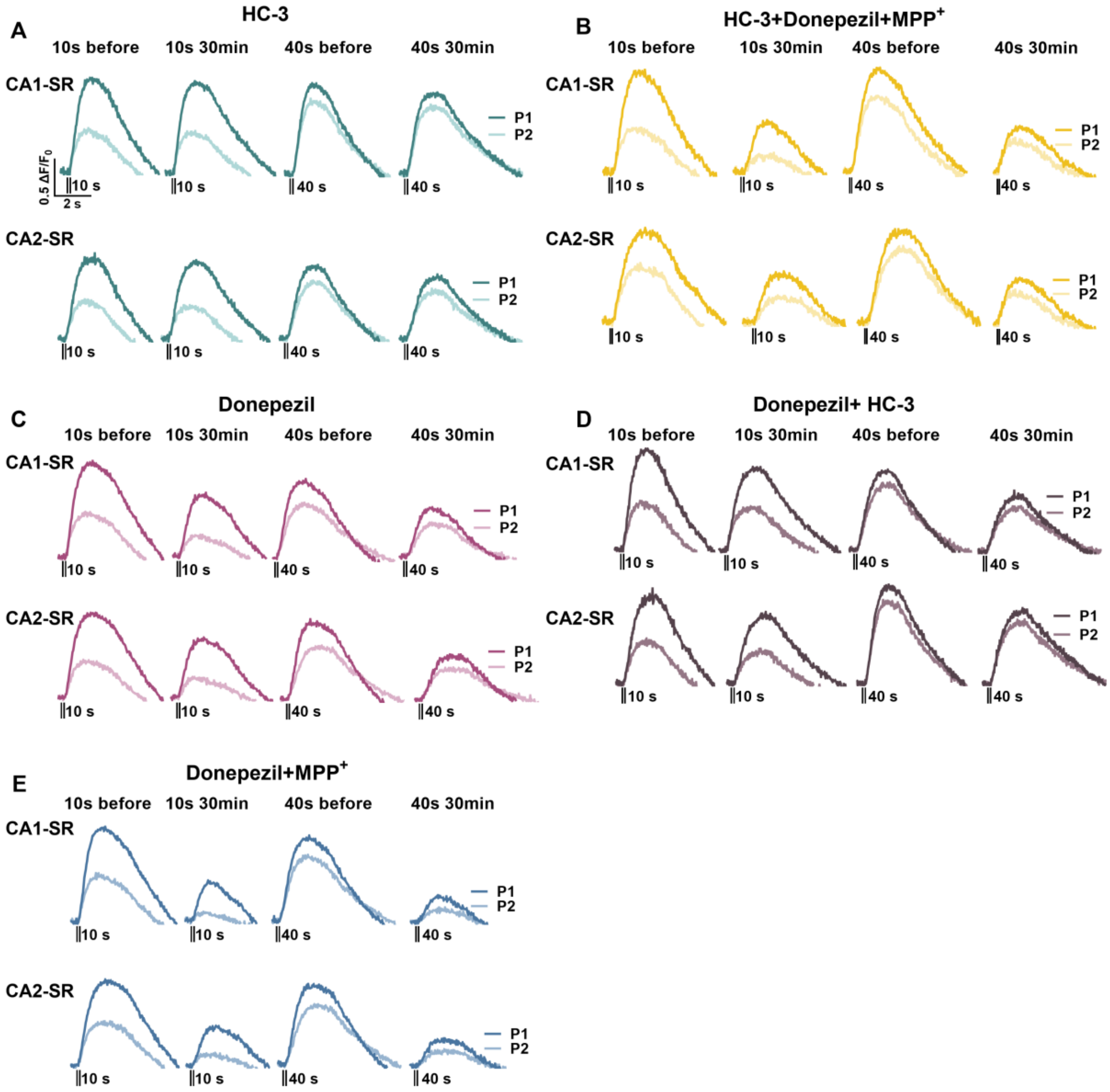

Supplementary Figure 10. AchE antagonist donepezil exacerbated MPP+-induced impairment of paired-burst dopamine release ratio in the CA1-SR and CA2-SR regions. (A) Representative fluorescence traces showing the response to paired-burst stimulation with inter-burst intervals of 10 s and 40 s before and after a 30-minute application of 15 μM HC-3 in CA1-SR and CA2-SR regions. **(B)** Representative fluorescence traces showing the response to paired-burst stimulation with inter-burst intervals of 10 s and 40 s before and after a 30-minute application of 15 μM HC-3, 50 μM MPP+, and 10 μM donepezil in CA1-SR and CA2-SR regions. **(C)** Representative fluorescence traces showing the response to paired-burst stimulation with burst intervals of 10 s and 40 s before and after a 30-minute application of 10 μM donepezil in CA1-SR and CA2-SR regions. **(D)** Representative fluorescence traces in response to paired-burst stimulation with 10 s and 40 s inter-burst intervals before and after a 30-minute co-application of 15 μM HC-3 and 10 μM donepezil in CA1-SR and CA2-SR regions. **(E)** Representative fluorescence traces showing the response to paired-burst stimulations before and after a 30-minute application of MPP+ and 10 μM donepezil in CA1-SR and CA2-SR regions (see summary in Figure 13).

Supplementary Table 1. List of primers used in genotyping

**Name**	**Sequence**
Transgene Forward	5’-GCTAACCATGTTCATGCCTTC-3’
Transgene Reverse	5’-AGGCAAATTTTGGTGTACGG-3’
Internal Positive Control Forward	5’-CAAATGTTGCTTGTCTGGTG-3’
Internal Positive Control Reverse	5’-GTCAGTCGAGTGCACAGTTT-3’
Ask1 FLOX Forward	5-AATTGGGCGAAGCTAAGCCACTTTTG-3′
Ask1 FLOX Reverse	5-CTGAGCCCAGAAAGCGAAGGA-3′

Supplementary Table 2. List of primers used in qPCR amplification reactions

**Name**	**Sequence**
Gapdh-Forward	5’- AGGTCGGTGTGAACGGATTTG-3’
Gapdh-Reverse	5’- TGTAGACCATGTAGTTGAGGTCA-3’
Ask1-Forward	5’- CCATCTTGGAGTGCGAGAA-3’
Ask1-Reverse	5’- GGACTGGAGTGAATCGGAAT-3’
Slc5a7-Forward	5’- CCTGCACTGATGGGAGAGAT -3’
Slc5a7-Reverse	5’- GGCAATGAGTGCAGAGACAA -3’
Srp54b-Forward	5’-CTGGTTTCTGGACCGCC-3’
Srp54b-Reverse	5’-TCACGGAGTTCCCCATTTCA-3’

Depdc1b-Forward	5'- TTGAGGACATCAAGGGGAAG -3'
Depdc1b-Reverse	5'- TGATACAGGGGCTTCTTTGG -3'
H2-q9-Forward	5'-CTGGTTTCTGGACCGCC-3'
H2-q9- Reverse	5'-CTGGTTTCTGGACCGCC-3'
